# On the Use of Aggregate Survey Data for Estimating Regional Major Depressive Disorder Prevalence

**DOI:** 10.1007/s11336-021-09808-8

**Published:** 2021-09-06

**Authors:** Domingo Morales, Joscha Krause, Jan Pablo Burgard

**Affiliations:** 1 Operations Research Center, University Miguel Hernández de Elche, Elche, Spain; 2 Department of Economic and Social Statistics, Trier University, Trier, Germany

**Keywords:** empirical best prediction, generalized linear mixed model, method of moments, parametric bootstrap, small area estimation

## Abstract

Major depression is a severe mental disorder that is associated with strongly increased mortality. The quantification of its prevalence on regional levels represents an important indicator for public health reporting. In addition to that, it marks a crucial basis for further explorative studies regarding environmental determinants of the condition. However, assessing the distribution of major depression in the population is challenging. The topic is highly sensitive, and national statistical institutions rarely have administrative records on this matter. Published prevalence figures as well as available auxiliary data are typically derived from survey estimates. These are often subject to high uncertainty due to large sampling variances and do not allow for sound regional analysis. We propose a new area-level Poisson mixed model that accounts for measurement errors in auxiliary data to close this gap. We derive the empirical best predictor under the model and present a parametric bootstrap estimator for the mean squared error. A method of moments algorithm for consistent model parameter estimation is developed. Simulation experiments are conducted to show the effectiveness of the approach. The methodology is applied to estimate the major depression prevalence in Germany on regional levels crossed by sex and age groups.

**Supplementary Information:**

The online version supplementary material available at 10.1007/s11336-021-09808-8.

## Introduction

Major depressive disorder (MDD) is a very prevalent and severe mental disorder that is associated with significantly shortened life expectancy (Walker et al., 2015; Gilman et al., 2017). Empirical studies found that it may cause up to $50\%$ increase in mortality risk relative to non-depressed individuals (Cuijpers et al., 2014). The effect is evident in both short-term and long-term survival rates, due to unnatural causes of death like suicide on the one hand (Zivin et al., 2015), and chronic conditions on the other hand (Glymour et al., 2010; Kozela et al., 2016). See Gilman et al. (2017) for a comprehensive overview. Moreover, empirical studies suggest that both the prevalence and the negative impact of depressive disorders varies geographically, as, for instance, reported by Haan et al. (2019) and Brignone et al. (2020). Against this background, assessing MDD on regional levels marks an important indicator for public health reporting. It allows policy-makers to plan comprehensive health care programs. Moreover, it may facilitate further explorative studies regarding the impact of living conditions on depression, which may improve our understanding of these disorders.

However, quantifying the regional prevalence of MDD is methodologically challenging. The topic is highly sensitive and national statistical institutions rarely have administrative records on this matter. Published figures are typically direct estimates that are produced based on survey data. In Germany, there are two major surveys that collect data on public health: *Sozio-oekonomisches Panel* (SOEP; Wagner et al., 2007) and *Gesundheit in Deutschland aktuell* (GEDA; Lange et al., 2015). The SOEP is a panel survey that is collected annually via computer-assisted personal interviews and questionnaires. In 2011, it contained 20828 individuals of age 18 or older. GEDA, on the other hand, is a telephone survey that is collected in irregular intervals. In 2010, it covered 22050 individuals of age 18 or older. Note that in 2011, the survey was not conducted. The amount of resources used to collect these samples is substantial. However, despite the considerable effort, the obtained sample data still lack in geographic detail. Since the German population is more than 80 million, the achieved sample sizes in both surveys are insufficient to provide reliable estimates on regional levels. The direct survey-based estimates suffer from large sampling variances and do not allow for the analysis of local MDD patterns. Thus, alternative statistical approaches based on auxiliary data are required to estimate the MDD prevalence.

Small area estimation (SAE) solves this problem by combining auxiliary data from multiple regions within a suitable regression model. Prevalence estimates are then obtained via model prediction. See Pfeffermann (2013), Rao and Molina (2015), Sugasawa and Kubokawa (2020), or Morales et al. (2021) for a comprehensive overview on SAE methods. Statistical modeling plays an essential role in SAE. Models can be designed to describe either unit-level data, or records that are aggregated by territories or population subgroups, which is called area-level data. In the first case, the fitted model is used to predict the values of the target variable in the unsampled part of the population. These predictions are subsequently used to construct estimators of regional prevalence and other population parameters of interest. However, it has the disadvantage of requiring information external to the survey, like the population means of the auxiliary variables or even a census file. In the second case, aggregated data modeling allows borrowing information from other domains, auxiliary variables from external data sources, as well as correlation structures. This allows the researcher to introduce model-based prevalence predictors that are more precise than direct estimators. The area-level approach has fewer restrictions to incorporate auxiliary variables and can be used without the need to use confidential individual data. This makes it more flexible and applicable in the field of psychometrics. Against this background, we propose to use an area-level approach to estimate the regional MDD prevalence.

In general, SAE allows for an efficiency advantage over the previously mentioned direct MDD prevalence estimates when the regression model contains sufficient predictive power. This is the case when (i) the chosen model fits the distribution characteristics of the target variable, and (ii) the auxiliary data have explanatory power. Regarding the first aspect, since prevalence figures may be viewed as proportions or counts, binomial, negative binomial, and Poisson mixed models are well-studied options. They are special cases of the generalized linear mixed model (GLMM), as, for instance, discussed by Breslow and Clayton (1993) and applied to SAE problems by Jiang (2003), Ghosh and Maiti (2004), Sugasawa et al. (2017), Hobza et al. (2020) and Faltys et al. (2020). The binomial logit approach for the estimation of regional proportions has been used by Molina et al. (2007), Ghosh et al. (2009), Chen and Lahiri (2012), Erciulescu and Fuller (2013), López-Vizcaíno et al. (2013), López-Vizcaíno et al. (2015), Militino et al. (2015), Chambers et al. (2016), Hobza and Morales (2016), Liu and Lahiri (2017), as well as Hobza et al. (2018). The Poisson or negative binomial mixed models were applied to estimate small area counts or proportions by Chambers et al. (2014), Dreassi et al. (2014), and Boubeta et al. (2016; 2017), among others.

Given these references, there are seemingly suitable SAE models for the estimation of the regional MDD prevalence in Germany. However, given the second aspect mentioned above, our auxiliary data situation prevents us from applying them. Using an area-level approach on SOEP and GEDA data requires the calculation of direct estimates for auxiliary variables to improve the MDD prevalence estimates. These records are subject to sampling errors, since the values have been estimated from survey samples. To ensure that SAE produces reliable results under these circumstances, the model must explicitly account for measurement errors in both the target variable and auxiliary variable records. This issue was first addressed under linear mixed models (LMMs) by Ybarra and Lohr (2008). They proposed an area-level model, where the continuous dependent variable is a direct estimator of a domain characteristic, and the covariates are direct estimators calculated from a different survey. They introduced a generalization of the Fay-Herriot model to covariates measured with error. As the auxiliary variables are direct estimators, their variance matrix is estimated with a design-based estimator applied to the corresponding unit-level data. The considered area-level model contains measurement errors with known variance matrices, but without specifying its distribution. The authors derived predictors of domain parameters and proposed estimators of the corresponding mean squared errors.

The literature about SAE methodology based on measurement error mixed model contains further interesting contributions. Ghosh and Sinha (2007), Torabi et al. (2009), as well as Singh (2011) introduced empirical Bayes predictors of finite population means under measurement error models. Torabi (2013) presented an application of data cloning to the estimation of the parameters of a measurement error nested error regression model. Singh (2011) applied the simulation extrapolation (SIMEX), ordinary corrected scores and Monte Carlo corrected scores methods of bias correction in the Fay-Herriot model, and investigated the performance of the bias-corrected estimators. Marchetti et al. (2015) presented a Big Data application of a measurement error Fay-Herriot model to estimate poverty indicators. Burgard et al. (2020) and Burgard et al. (2021) considered structural versions of the univariate and multivariate Fay-Herriot models. Bell et al. (2019) compared functional, structural, and naïve area-level models in the SAE setup. Concerning the Bayesian approach, Ghosh et al. (2006) introduced hierarchical Bayes predictors of finite population parameters under structural measurement error models. Arima et al. (2015) introduced a measurement error hierarchical model and derived Bayes predictors. Arima et al. (2017) proposed multivariate Fay-Herriot Bayesian predictors of small area means. Arima and Polettini (2019) considered a Bayesian unit-level small area model with misclassified covariates.

The above-cited studies proposed extensions to SAE models based on continuous target variables to account for measurement errors. However, recall that MDD prevalence estimation requires proportions or counts as target variable, which have to be modeled with GLMMs. Unfortunately, there currently no suitable measurement error extensions for GLMMs that could be applied to the SOEP and GEDA. Accordingly, new statistical methodology has to be developed to close this gap. This can be done in two ways, depending on whether frequentist or Bayesian procedures developed for LMMs are adapted to GLMMs. This papers follows the frequentist approach and leaves open the study of Bayesian statistical methodologies.

We propose a new area-level Poisson mixed model that is capable of accounting for measurement errors in covariates. With this feature, it is a GLMM for SAE that allows for robust predictions from uncertain auxiliary data that is measured with error. A detailed model description is provided and empirical best predictors (EBP) based on the model are derived. We further state a parametric bootstrap estimator of the mean squared error (MSE) of prediction. A consistent method of moments algorithm for model parameter estimation is presented. Further, it is demonstrated how the variances of the model parameter estimates can be approximated. Simulation experiments are conducted in order to establish the effectiveness of the approach. The new methodology is subsequently applied for the estimation of the regional MDD prevalence in Germany within the year 2011. For the target variable, we use area-level survey records from the SOEP. For the auxiliary data, we consider direct area-level estimates obtained from GEDA.

The remainder of the paper is organized as follows. Section 2 introduces the problem of interest and describes the data employed in the estimation of regional prevalence. Section 3 presents the new model, derives EBPs, and proposes a parametric bootstrap estimator of the MSEs. Section 4 states the method of moments (MM) for model parameter estimation and elaborates on variance approximation. Section 5 contains the simulation experiments. Section 6 presents the application of the model-based statistical methodology to the data from the German surveys SOEP and GEDA. Section 7 closes with some conclusive remarks. The paper is supported by a supplemental material file containing three appendices. Appendix A gives the components of the Newton–Raphson algorithm that calculates the MM estimators of the model parameters. Appendix B provides developments that establish consistency of the MM estimators. Appendix C presents additional simulation experiments.

## The Problem of Interest and the Data

The problem that motivates the research on new statistical methodology is the regional prevalence estimation of MDD in Germany. For regions with big sample size, we can carry out the estimation by means of direct estimators. However, if domain sample sizes are not big enough then direct estimators are not precise. This problem can be addressed by reinforcing the direct information provided by the target variable with the indirect information provided by correlated auxiliary variables or data from other domains. In what follows, we describe the survey data employed in the estimation of regional prevalence.

The base population are the citizens of Germany that are 18 years or older from the year 2011. The domains are defined as cross combinations of (i) the seven Nielsen regions of the country, (ii) binary sex, as well as (iii) the age groups I: 18-44, II: 45-64, III: 65+. There are $D=42$ domains, and we are interested in estimating the domain prevalences

$$\begin{aligned} {\bar{Y}}_d = \frac{1}{N_d} \sum _{j \in U_d} y_{dj}, \quad y_{dj} = {\left\{ \begin{array}{ll} 1, &{} j \ \text {reports to have a MDD} \\ 0, &{} \text {else} \end{array}\right. }, \quad d=1, \ldots , D, \end{aligned}$$

where $N_d$ is the domain size and *i* denotes an individual in domain $U_d$. In accordance with the developments in Sect. 3, two data sources are required to implement the model.

For the target variable, we use the 2011 wave of the German survey SOEP with 20828 individuals (Wagner et al., 2007). Please note that the SOEP originally covers citizens that are 16 years or older. However, we have removed individuals of age 16 and 17 from the sample in order to obtain a coherent population for both target variable and auxiliary data. The domain-specific sample sizes range from 278 to 840. See Goebel et al. (2008) for further details on the survey design, interview mode, and data processing in SOEP. The survey mainly covers socioeconomic topics, but also assesses medical conditions and other health-related information. This includes several mental disorders. Against this background, we define the sample counts $y_d$, $d=1, \ldots , D$, as the number of sampled individuals per domain that reported being diagnosed with MDD in SOEP. The sample counts range from 5 to 84.

For the auxiliary data, we use the 2010 sample of the German health survey GEDA (Lange et al., 2015). Note that in 2011, the survey was not conducted. The sample contains citizens of age 18 and older from the German population that are interviewed via computer-assisted telephone interviews in a representative random sample. The data set contains a broad range of health-related information of participants on the person level. For further information on the survey as well as its sampling design and response rates, see Robert Koch Institute (2013). The GEDA sample size for 2010 is 22050, which is larger than the SOEP sample, which allows using estimates of the first survey as auxiliary data for the second one. Further, we note that GEDA and SOEP are independent surveys. That is, there is no sampling error covariance between the target variable stemming from the SOEP and the covariates stemming from GEDA. If the target variable and the covariates stem from the same survey, the error covariance structure has to be taken into account additionally. This is not covered by the presented methodology and would lead to a new topic for research. In order to select a statistical model for explaining the behavior of the target variable MDD, we have to select a set of covariates from GEDA that explain the variation of the sample counts of individuals having MDD. The selection is based on methodological considerations on the one hand, and on related literature concerning MDD on the other hand. All in all, we consider the subsequent covariates:

- *Domain size:* Number of individuals in domain $U_d$ (scaled)
- *Sex:* Binary sex (male, female) that domain $U_d$ is associated with
- *Depr. treatment:* Share of people that have been treated for depression in $U_d$
- *SES score:* Average score of the socioeconomic status in $U_d$

The variable *Domain size* is obtained from administrative records of the German census 2011. It is included as a more flexible substitute for an intercept in order to stabilize the fitting behavior. The equations of the MM algorithm are based on differences between theoretical moments under a given parametrization and observed sample moments. In the presence of measurement errors, an objective function based on these differences is much more flat relative to situations where values are measured correctly. Including the domain size as fixed effect reduces the variability of the exponent in the exponential function and leads to a numerically more stable estimation process. However, we have scaled the respective values in order to avoid computational infinities arising from the usage of the exponential function in the statistic software R. We let $N_d := |U_d|$ and define the value of *Domain size* for domain $U_d$ as $x_{d} = N_d / \delta$, where $\delta$ is a scalar that is constant over all domains. We choose $\delta = (\sum _{d=1}^D N_d)/ (\sum _{d=1}^D n_d)$ in order to obtain values of the same magnitude as the domain sample sizes of the SOEP for 2011.

The binary auxiliary variable *Sex* is included because the domains are constructed as cross combinations of age, region, and sex. It is well known from the literature that females tend to have a higher probability to be diagnosed with major depression than males. See Cyranowski et al. (2000) and Albert (2015) for further details. Accordingly, we expect female domains to show higher depression proportions in the sample than male domains. The variable *Depr. treatment* is an obvious choice given its content. A high share of people that are treated for depression is likely to be associated with an increased number of depressed individuals. Furthermore, the sample counts and *Depr. treatment* showed a reasonably positive correlation with 0.3. And finally, the variable *SES score* is a combined metric that is based on education, income, and professional position. See Robert Koch Institute (2013) for more specific descriptions. Empirical studies found to that the socioeconomic status is negatively associated with depressive disorders. Thus, we expect high socioeconomic status to be associated with lower depression proportions. However, note that there is debate in the literature with regards to which aspect of the status actually drives this relation. For further details, we refer to Jo et al. (2011) and Freeman et al. (2016).

## Model

### Formulation

This section introduces the area-level measurement error Poisson mixed (MEPM) model. Let *U* denote a finite population that is segmented into *D* domains indexed by $d=1, \ldots , D$. We assume a situation where (i) survey data for a variable of interest *y*, and (ii) either administrative records or survey data regarding auxiliary variables $\varvec{x}= \{x_1, \ldots , x_p\}$ are available. The area-level MEPM model is composed of three hierarchical stages. In the first stage a model, called *sampling* model, gives the distribution of the sample counts $y_d$. Let $p_d$ be the domain probability of an event of interest and let $\nu _d=n_d$ be the domain sample size (or a size parameter in other counting setups). The sampling model indicates that the distribution of the target variable $y_{d}$ is

$$\begin{aligned} y_{d}\sim \text{ Poiss }(\nu _{d}p_{d}),\quad d=1,\ldots ,D. \end{aligned}$$

That is to say, the target variable $y_d$ gives the observed domain counts in the sample for the event of interest. Against the background of Sect. 2, $y_d$ refers to the number of individuals in the sample that have a MDD in domain *d*. In the second stage, the logarithm of the mean parameter $\mu _d=\nu _dp_d$ (the natural parameter in Poisson regression models) is assumed to vary linearly with the *p* area-level auxiliary variables contained in $\varvec{x}$. In the second stage, we consider a set of random effects $\{v_{d}:\,\,d=1,\ldots ,D\}$ that are independent and identically distributed (i.i.d.) according to $v_d \sim N(0,1)$. In matrix notation, we have

$$\begin{aligned} \varvec{v}=\underset{1\le d \le D}{\text{ col }}(v_{d})\sim N_D(\varvec{0},\varvec{I}_D), \quad f_v(\varvec{v})=(2\pi )^{-D/2}\exp \left\{ -\frac{1}{2}\,\varvec{v}^\prime \varvec{v}\right\} , \end{aligned}$$

where $f_v(\varvec{v})$ gives the probability density function of $\varvec{v}$. The *linking* model is specified as

$$\begin{aligned} \log \mu _{d}=\log \nu _{d}+\log p_{d}=\log \nu _{d}+ {{\tilde{\varvec{x}}}}_{0,d}\varvec{\beta }_0+{{\tilde{\varvec{x}}}}_{1,d}\varvec{\beta }_1+\phi v_{d},\quad d=1,\ldots ,D, \end{aligned}$$

where ${{\tilde{\varvec{x}}}}_{0,d}$ and ${{\tilde{\varvec{x}}}}_{1,d}$ are a $1\times p_1$ and $1\times p_2$ row vectors, respectively, containing the true aggregated values of $p=p_1+p_2$ auxiliary variables for domain *d*. The terms

$$\begin{aligned} \varvec{\beta }_0=\underset{1\le k \le p_1}{\hbox {col}}(\beta _{k}), \quad \varvec{\beta }_1=\underset{p_1+1\le k \le p}{\hbox {col}}(\beta _{k}), \end{aligned}$$

are the corresponding column vectors of regression parameters. By defining ${{\tilde{\varvec{x}}}}_{d}=({{\tilde{\varvec{x}}}}_{0,d},{{\tilde{\varvec{x}}}}_{1,d})$ and $\varvec{\beta }=(\varvec{\beta }_0^\prime ,\varvec{\beta }_1^\prime )^\prime$, the linking model can be written in the simpler form.

$$\begin{aligned} \log \mu _{d}=\log \nu _{d}+\log p_{d}=\log \nu _{d}+ {{\tilde{\varvec{x}}}}_{d}\varvec{\beta }+\phi v_{d},\quad d=1,\ldots ,D. \end{aligned}$$

We assume that ${{\tilde{\varvec{x}}}}_{0,d}$ is known and equal to $\varvec{x}_{0,d}$, $d=1,\ldots ,D$. With respect to practice, $\varvec{x}_{0,d}$ may correspond to administrative records that are taken from a register. Further, we assume that the ${{\tilde{\varvec{x}}}}_{1,d}$’s are unknown random vectors with predictors $\varvec{x}_{1,d}$’s calculated from independent data sources. These data sources might be data sets obtained from surveys with larger sample sizes than the survey where the dependent variables $y_d$’s are recorded. In the third stage, we consider the *measurement error* model

1 $$\begin{aligned} {{\tilde{\varvec{x}}}}_{1,d}^\prime =\varvec{x}_{1,d}^\prime +\varvec{u}_d,\quad \varvec{u}_d\sim N_{p_1}(\varvec{0}, \varvec{\Sigma }_d),\quad d=1,\ldots ,D, \end{aligned}$$

where $\varvec{x}_{1,d}$ is a row vector containing unbiased predictors of the components of ${{\tilde{\varvec{x}}}}_{1,d}$, $\varvec{u}_d$ is a column vector of random measurement errors, $\varvec{\Sigma }_d$ is the known covariance matrix of $\varvec{u}_d$, and $\varvec{u}_d$ and $\varvec{x}_{1,d}$, $d=1,\ldots ,D$, are independent. In practice, $\varvec{\Sigma }_d$ is typically unknown and subsequently estimated from the same unit-level survey data as $\varvec{x}_{1,d}$. We finally assume that $\varvec{x}_{1,d}$, $\varvec{u}_d$, $v_d$, $d=1,\ldots ,D$, are independent, but we only introduce inference procedures conditionally on $\varvec{x}_{d}=(\varvec{x}_{0,d},\varvec{x}_{1,d})$, $d=1,\ldots ,D$. In matrix notation, we have

$$\begin{aligned} \varvec{y}=\underset{1\le d \le D}{\text{ col }}(y_{d}),\quad \varvec{X}=\underset{1\le d \le D}{\hbox {col}}(\varvec{x}_{d}),\quad \varvec{u}=\underset{1\le d \le D}{\text{ col }}(\varvec{u}_{d}), \quad \varvec{u}_d=\underset{1\le k \le p_1}{\text{ col }}(u_{dk}). \end{aligned}$$

Based on these definitions, the probability density function of the measurement errors is

$$\begin{aligned} f_u(\varvec{u})=(2\pi )^{-Dp_1/2}\bigg (\prod _{d=1}^D|\varvec{\Sigma }_{d}|\bigg )^{-1/2} \exp \bigg \{-\frac{1}{2}\,\sum _{d=1}^D\varvec{u}_d^\prime \varvec{\Sigma }_d^{-1}\varvec{u}_d\bigg \}. \end{aligned}$$

The area-level MEPM model can be expressed as a single model in the form

2 $$\begin{aligned} y_{d}|_{v_{d},\varvec{u}_d,\varvec{x}_d}\sim \text{ Poiss }(\nu _{d}p_{d}),\,\, \log \mu _{d}=\log \nu _{d}+\log p_{d}=\log \nu _{d}+\varvec{x}_d\varvec{\beta }+\varvec{u}_{d}^\prime \varvec{\beta }_1+\phi v_{d}, \end{aligned}$$

$p_{d}=\exp \left\{ \varvec{x}_{d}\varvec{\beta }+\varvec{u}_{d}^\prime \varvec{\beta }_1+\phi v_{d}\right\}$, $d=1,\ldots ,D$. From the model assumptions, it holds that

$$\begin{aligned}&P(y_{d}|\varvec{v},\varvec{u},\varvec{X})=P(y_{d}|v_d,\varvec{u}_d,\varvec{x}_d)=\frac{1}{y_{d}!}\exp \{-\nu _{d}p_{d}\} \nu _{d}^{y_{d}} p_{d}^{y_{d}}, \\&P(\varvec{y}|\varvec{v},\varvec{u},\varvec{X})=\prod _{d=1}^DP(y_{d}|v_d,\varvec{u}_d,\varvec{x}_d), \\&P(\varvec{y}|\varvec{X})=\int _{R^{D}}\int _{R^{Dp_1}} P(\varvec{y}|v_d,\varvec{u}_d,\varvec{x}_d) f_{v}(\varvec{v})f_u(\varvec{u})\,d\varvec{v}\,d\varvec{u}=\int _{R^{D(p_1+1)}} \psi (\varvec{y},\varvec{v},\varvec{u})\,d\varvec{v}\,d\varvec{u}, \end{aligned}$$

where

$$\begin{aligned} \psi (\varvec{y},\varvec{v},\varvec{u})= & {} (2\pi )^{-\frac{D}{2}}\exp \left\{ \frac{-\varvec{v}^\prime \varvec{v}}{2}\right\} (2\pi )^{-Dp_1/2}\bigg (\prod _{d=1}^D|\varvec{\Sigma }_{d}|\bigg )^{-1/2}\exp \bigg \{-\frac{1}{2}\,\sum _{d=1}^D\varvec{u}_d^\prime \varvec{\Sigma }_d^{-1}\varvec{u}_d\bigg \} \\&\cdot&\prod _{d=1}^D\frac{\exp \{-\nu _{d}p_{d}\} \nu _{d}^{y_{d}} \exp \left\{ y_{d}(\varvec{x}_{d}\varvec{\beta }+\varvec{u}_{d}^\prime \varvec{\beta }_1+\phi v_{d})\right\} }{y_{d}!} \\= & {} (2\pi )^{-\frac{D}{2}}\exp \left\{ \frac{-\varvec{v}^\prime \varvec{v}}{2}\right\} (2\pi )^{-Dp_1/2}\bigg (\prod _{d=1}^D|\varvec{\Sigma }_{d}|\bigg )^{-1/2}\exp \bigg \{-\frac{1}{2}\,\sum _{d=1}^D\varvec{u}_d^\prime \varvec{\Sigma }_d^{-1}\varvec{u}_d\bigg \} \\&\cdot&\big (\prod _{d=1}^Dy_{d}!\big )^{-1} \exp \left\{ \sum _{d=1}^D\big \{-\nu _{d}\exp \{\varvec{x}_{d}\varvec{\beta }+\varvec{u}_{d}^\prime \varvec{\beta }_1+\phi v_{d}\}+y_{d}\log \nu _{d}\big \}\right\} \\&\cdot&\exp \left\{ \sum _{k=1}^p\big (\sum _{d=1}^Dy_{d}x_{dk}\big )\beta _k+\sum _{k=1}^{p_1}\big (\sum _{d=1}^Dy_{d}u_{dk}\big )\beta _{ k} +\phi \sum _{d=1}^Dy_{d}v_{d}\right\} . \end{aligned}$$

### Empirical Best Prediction

This section derives the EBPs of the domain probability $p_d$ and the mean parameter $\mu _d = \nu _d p_d$. We proceed according to the following basic strategy. First, the best predictors (BPs) are calculated under the model under the preliminary assumption that the model parameters $\varvec{\theta }= (\varvec{\beta }', \phi )'$ are known. Afterward, $\varvec{\theta }$ is replaced by a consistent estimator ${\hat{\varvec{\theta }}} = ({\hat{\varvec{\beta }}}', {\hat{\phi }})'$ to obtain the EBP. We show how to perform consistent model parameter estimation in Sect. 4. We start with the EBP of the domain probability $p_d$. Recall that the conditional distribution of $\varvec{y}$, given $\varvec{v}$, $\varvec{u}$ and $\varvec{X}$, is $P(\varvec{y}|\varvec{v},\varvec{u},\varvec{X})=\prod _{d=1}^D P(y_{d}|v_{d},\varvec{u}_d,\varvec{x}_d)$, where

$$\begin{aligned} P(y_{d}|v_{d},\varvec{u}_d,\varvec{x}_d)= & {} \frac{1}{y_{d}!}\exp \{-\nu _{d}p_{d}\} \nu _{d}^{y_{d}} p_{d}^{y_{d}} \\= & {} \frac{\nu _d^{y_d}}{y_d!} \exp \left\{ y_{d}(\varvec{x}_{d}\varvec{\beta }+\varvec{u}_{d}^\prime \varvec{\beta }_1+\phi v_d)-\nu _{d}\exp \{\varvec{x}_{d}\varvec{\beta }+\varvec{u}_{d}^\prime \varvec{\beta }_1+\phi v_{d}\}\right\} . \end{aligned}$$

The probability density functions of $v_d$ and $\varvec{u}_d$ are

$$\begin{aligned} f(v_d)=(2\pi )^{-1/2}\exp \big \{-\frac{1}{2}\, v_{d}^2\big \},\quad f(\varvec{u}_d)=(2\pi )^{-p_1/2}|\varvec{\Sigma }_{d}|^{-1/2} \exp \Big \{-\frac{1}{2}\,\varvec{u}_d^\prime \varvec{\Sigma }_d^{-1}\varvec{u}_d\Big \}. \end{aligned}$$

The BP of $p_{d}$ is its conditional expectation under the model, that is, ${\hat{p}}_{d}(\varvec{\theta })=E_\theta [p_{d}|\varvec{y},\varvec{X}]$. In this case, we have that $E_\theta [p_{d}|\varvec{y},\varvec{X}]=E_\theta [p_{d}|y_{d},\varvec{x}_d]$ and

$$\begin{aligned} E_\theta [p_{d}|y_{d},\varvec{x}_d]= & {} \frac{ \int _{R^{p_1+1}}\exp \{\varvec{x}_{d}\varvec{\beta }+\varvec{u}_{d}^\prime \varvec{\beta }_1+\phi v_{d}\} P(y_{d}|v_{d},\varvec{u}_d,\varvec{x}_d)f(v_{d})f(\varvec{u}_d)\,\mathrm{d}v_{d} \mathrm{d}\varvec{u}_{d}}{\int _{R^{p_1+1}}P(y_{d}|v_{d},\varvec{u}_d,\varvec{x}_d)f(v_{d})f(\varvec{u}_d)\,\mathrm{d}v_{d}\mathrm{d}\varvec{u}_{d}}\\= & {} \frac{N_{d}(y_{d},\varvec{\theta })}{D_{d}(y_{d},\varvec{\theta })}. \end{aligned}$$

The numerator and denominator of the fraction are given by

$$\begin{aligned} N_{d}(y_{d},\varvec{\theta })= & {} \int _{R^{p_1+1}}\exp \Big \{(y_{d}+1)(\varvec{x}_{d}\varvec{\beta }+\varvec{u}_{d}^\prime \varvec{\beta }_1+\phi v_{d}) - \nu _{d}\exp \big \{\varvec{x}_{d}\varvec{\beta }+\varvec{u}_{d}^\prime \varvec{\beta }_1+\phi v_{d}\big \}\Big \}\\&\quad \times f(v_{d})f(\varvec{u}_d)\,\mathrm{d}v_{d}\mathrm{d}\varvec{u}_{d}, \\ D_{d}(y_{d},\varvec{\theta })= & {} \int _{R^{p_1+1}}\exp \Big \{y_{d}(\varvec{x}_{d}\varvec{\beta }+\varvec{u}_{d}^\prime \varvec{\beta }_1+\phi v_{d}) - \nu _{d}\exp \{\varvec{x}_{d}\varvec{\beta }+\varvec{u}_{d}^\prime \varvec{\beta }_1+\phi v_{d}\}\Big \}\\&\quad \times f(v_{d})f(\varvec{u}_d)\,\mathrm{d}v_{d}\mathrm{d}\varvec{u}_{d}. \end{aligned}$$

In principle, the EBP is then calculated according to ${\hat{p}}_d = N_d(y_d, {\hat{\varvec{\theta }}})/ D_d(y_d, {\hat{\varvec{\theta }}})$. However, both integrals contained in $E_\theta [p_{d}|y_{d},\varvec{x}_d]$ do not have a closed-form solution. As a consequence, the EBP cannot be calculated exactly, but has to be approximated instead. This can be done by using Monte Carlo integration. That is to say, we choose a number of 2*L* symmetric locations at which numerator and denominator are evaluated within the support of the multivariate probability density of both random effects and measurement errors. Afterward, we average the obtained functional values and obtain an approximation to the corresponding integrals. This is summarized in the subsequent procedure.

1. Estimate $${\hat{\varvec{\theta }}}=({{\hat{\varvec{\beta }}}}',{\hat{\phi }})$$.
2. Generate $$v_{d}^{(\ell )}$$ i.i.d. *N*(0, 1), $$\varvec{u}_{d}^{(\ell )}$$ i.i.d. $$N_{p_1}(\varvec{0},\varvec{\Sigma }_{d})$$, $$v_{d}^{(L+\ell )}=-v_{d}^{(\ell )}$$, $$\varvec{u}_{d}^{(L+\ell )}=-\varvec{u}_{d}^{(\ell )}$$, $$d=1,\ldots ,D$$, $$\ell =1,\ldots ,L$$.
3. Calculate $${\hat{p}}_{d}({{\hat{\varvec{\theta }}}})={\hat{N}}_{d}/{\hat{D}}_{d}$$, where
  $$\begin{aligned} {\hat{N}}_{d}= & {} \frac{1}{2L}\sum _{\ell =1}^{2L}\exp \left\{ (y_{d}+1) (\varvec{x}_{d}{{\hat{\varvec{\beta }}}}+\varvec{u}_{d}^{\prime (\ell )}{{\hat{\varvec{\beta }}}}+{{\hat{\phi }}} v_{d}^{(\ell )}) - \nu _{d}\exp \{\varvec{x}_{d}{{\hat{\varvec{\beta }}}}+\varvec{u}_{d}^{\prime (\ell )}{{\hat{\varvec{\beta }}}}+{{\hat{\phi }}} v_{d}^{(\ell )}\}\right\} , \\ {\hat{D}}_{d}= & {} \frac{1}{2L}\sum _{\ell =1}^{2L}\exp \left\{ y_{d}(\varvec{x}_{d}{{\hat{\varvec{\beta }}}}+\varvec{u}_{d}^{\prime (\ell )}{{\hat{\varvec{\beta }}}}+{{\hat{\phi }}} v_{d}^{(\ell )}) - \nu _{d}\exp \{\varvec{x}_{d}{{\hat{\varvec{\beta }}}}+\varvec{u}_{d}^{\prime (\ell )}{{\hat{\varvec{\beta }}}}+{{\hat{\phi }}} v_{d}^{(\ell )}\}\right\} . \end{aligned}$$

It can be shown that for $L \rightarrow \infty$, it holds that ${\hat{N}}_{d} / {\hat{D}}_{d} \overset{a.s.}{\rightarrow } N_d(y_d, {\hat{\varvec{\theta }}})/ D_d(y_d, {\hat{\varvec{\theta }}})$ (Calfisch, 1998). That is to say, for *L* chosen sufficiently large, the upper algorithm can approximate the EBP up to arbitrary order. Based on these developments, we can conclude that the EBP of the mean parameter $\mu _d=\nu _d p_d$ is ${\hat{\mu }}_{d}({{\hat{\varvec{\theta }}}})=\nu _d{\hat{p}}_{d}({{\hat{\varvec{\theta }}}})$. Note that there may be applications where the researcher wants to avoid Monte Carlo integration, for instance when $p_1$ is large and heavy computational burden shall be avoided. For such cases, we state synthetic predictors as an alternative that can be calculated very efficiently. The synthetic predictor of $p_d$ is ${\tilde{p}}_d^{syn}=\exp \{ \varvec{x}_{d}{\hat{\varvec{\beta }\}}}$. Likewise, the synthetic predictor of $\mu _d=\nu _d p_d$ is ${\tilde{\mu }}_d^{syn} = \nu _d \exp \{ \varvec{x}_{d}{\hat{\varvec{\beta }\}}}$. These predictors have acceptable accuracy when both measurement errors and random effects are negligible in the distribution of the target variable.

### Mean Squared Error Estimation

This section presents a parametric bootstrap algorithm that estimates the mean squared error (MSE) of the EBP ${\hat{\mu }}_d={\hat{\mu }}_d({\hat{\varvec{\theta }}})$, which is characterized by

$$\begin{aligned} MSE({\hat{\mu }}_d) = E \big [ ({\hat{\mu }}_d - \mu _d)^2 \big ], \quad d=1, \ldots , D. \end{aligned}$$

Let *B* be the number of bootstrap replicates. The procedure is given as follows.

1. Obtain estimates ${\hat{\varvec{\theta }}}=({\hat{\varvec{\beta }}}, {\hat{\phi }})'$ based on the observations $(y_1, \varvec{x}_1), \ldots , (y_D, \varvec{x}_D)$
2. For $$b=1, \ldots , B$$, do
  - Generate $$v_{d}^{(b)} \overset{i.i.d.}{\sim } N(0,1)$$, $$\varvec{u}_{d}^{(b)}\overset{i.i.d.}{\sim }N_p(\varvec{0},\varvec{\Sigma }_{d})$$, $$d=1, \ldots , D$$
  - Calculate $\mu _d^{(b)} = \nu _d p_d^{(b)}$, $p_d^{(b)} = \exp \{\varvec{x}_d{\hat{\varvec{\beta }}} + \varvec{u}_d^{(b)\prime }{\hat{\varvec{\beta }}}+{\hat{\phi }}v_d^{(b)}\}$, and draw $y_d^{(b)} \sim \text {Poiss}(\mu _d^{(b)})$, $d=1, \ldots , D$
  - Obtain estimates ${\hat{\varvec{\theta }}}^{(b)}=({\hat{\varvec{\beta }}}^{(b)}, {\hat{\phi }}^{(b)})$ based on the observations $(y_1^{(b)}, \varvec{x}_1), \ldots , (y_D^{(b)}, \varvec{x}_D)$
  - Calculate the EBP ${\hat{\mu }}_d^{(b)} = {\hat{\mu }}_d({\hat{\varvec{\theta }}}^{(b)})$, $d=1, \ldots , D$
3. Output: $$mse^*({\hat{\mu }}_d) = \frac{1}{B} \sum _{b=1}^B ({\hat{\mu }}_d^{(b)} - \mu _d^{(b)})^2$$

The MSE estimator based on this naive parametric bootstrap approach has generally $O(D^{-1})$ bias, which is not always acceptable in practice. More refinement can be achieved by double bootstrapping, as proposed Hall and Maiti (2006). We have not implemented the double bootstrap approach because it is computationally very intensive and has a great impact on simulation times. Nevertheless, in some real data setups with small *D* and high and accurate computing capacity, the double bootstrap approach is recommendable.

## Model Parameter Estimation

Conditioned to $\varvec{X}$, the model likelihood of the MEPM model, $P(\varvec{y}|\varvec{X})$, is an integral on $R^{D(p_1+1)}$. This fact is a drawback for estimating the model parameters by the maximum likelihood (ML) method. Therefore, we propose applying the MM method, which does not require approximating integrals. The MM method gives consistent estimators and it is computationally more efficient than the ML method. This section presents a Newton–Raphson algorithm to fit the area-level MEPM model via the MM approach. Note that computational details of the algorithm are located in Appendix A of the supplemental material. Further, Appendix B establishes the consistency of the MM estimators.

### Method of Moments

A natural set of equations for the MM approach is

3 $$\begin{aligned} 0= & {} f_k(\varvec{\theta })=M_k(\varvec{\theta })-{\hat{M}}_k=\sum _{d=1}^DE_\theta [y_{d}]x_{dk} -\sum _{d=1}^Dy_{d}x_{dk},\quad k=1,\ldots p, \end{aligned}$$

4 $$\begin{aligned} 0= & {} f_{p+1}(\varvec{\theta })=M_{p+1}(\varvec{\theta })-{\hat{M}}_{p+1}=\sum _{d=1}^DE_\theta [y_{d}^2]-\sum _{d=1}^Dy_{d}^2, \end{aligned}$$

where the model-based expectations $E_{\theta }[y_d]$ and $E_{\theta }[y_d^2]$ are given in Appendix A. For solving the system of nonlinear MM equations, we use a Newton–Raphson algorithm. Let $i=0,1,2, \ldots$ denote the index of iterations. Further, let $\varvec{f}(\varvec{\theta }^{(i)})$ be the set of MM equations based on the model parameter vector in the *i*-th iteration. Likewise, let $\varvec{H}(\varvec{\theta }^{(i)})$ denote the corresponding Jacobian matrix. The Newton–Raphson algorithm is performed as follows:

1. Set the initial values ${i}=0$ and $\varvec{\theta }^{(0)}=(\varvec{\beta }^{(0)},\phi ^{(0)})$.
2. Repeat the following steps till convergence
  - Update $\varvec{\theta }^{({i})}$ by using the equation
    $$\begin{aligned} \varvec{\theta }^{({i}+1)}=\varvec{\theta }^{({i})}-\varvec{H}^{-1}(\varvec{\theta }^{({i})})\varvec{f}(\varvec{\theta }^{({i})}), \end{aligned}$$
  - Update the iteration index ${i}\leftarrow {i}+1$.
3. Output: $${\hat{\varvec{\theta }}} = \varvec{\theta }^{({i}+1)}$$.

Appendix A demonstrates how to compute the components of the Jacobian matrix. Note that the MM estimator is consistent in probability, i.e., ${\hat{\varvec{\theta }}} \overset{P}{\rightarrow } \varvec{\theta }$ as $D \rightarrow \infty$. We proof this result in Appendix B based on the developments of Jiang (1998).

### Variance Estimation and Significance Testing

Hereafter, we show how to estimate the variance of the model parameter estimates obtained from the MM method. This step is important with respect to practice when significance tests for covariates that are measured with error shall be conducted. Let us define

$$\begin{aligned} \varvec{M}(\varvec{\theta })=\underset{1\le k \le p+1}{\hbox {col}}(M_k(\varvec{\theta })), \quad {{\hat{\varvec{M}}}}=\underset{1\le k \le p+1}{\hbox {col}}({\hat{M}}_k), \end{aligned}$$

where $M_k$ and ${\hat{M}}_k$ are defined as in Sect. 4.1. The asymptotic variance of the MM estimators can be approximated by a Taylor expansion of $\varvec{M}(\varvec{\theta })$ around $\varvec{\theta }$. This is to say,

$$\begin{aligned} {\hat{\varvec{M}}}=\varvec{M}({{\hat{\varvec{\theta }}}})\approx \varvec{M}(\varvec{\theta })+\varvec{H}(\varvec{\theta })({{\hat{\varvec{\theta }}}}-\varvec{\theta }),\quad {{\hat{\varvec{\theta }}}}-\varvec{\theta }\approx \varvec{H}^{-1}(\varvec{\theta })({{\hat{\varvec{M}}}}-\varvec{M}(\varvec{\theta })). \end{aligned}$$

Under some regularity conditions, it holds that

$$\begin{aligned} \text{ var }({{\hat{\varvec{\theta }}}})=E[({{\hat{\varvec{\theta }}}}-\varvec{\theta })({{\hat{\varvec{\theta }}}}-\varvec{\theta })^\prime ]\approx \varvec{H}^{-1}(\varvec{\theta })\text{ var }({{\hat{\varvec{M}}}})\varvec{H}^{-1}(\varvec{\theta }). \end{aligned}$$

An estimator of $\text{ var }({{\hat{\varvec{\theta }}}})$ is

$$\begin{aligned} \widehat{\text{ var }}({{\hat{\varvec{\theta }}}})=\varvec{H}^{-1}({{\hat{\varvec{\theta }}}})\widehat{\text{ var }}({{\hat{\varvec{M}}}})\varvec{H}^{-1}({{\hat{\varvec{\theta }}}}), \end{aligned}$$

where ${{\hat{\varvec{\theta }}}}=\varvec{\theta }^{(r+1)}$ is taken from the output of the MM algorithm and $\widehat{\text{ var }}({{\hat{\varvec{M}}}})$ is an estimator of the covariance matrix of ${{\hat{\varvec{M}}}}$. An algorithm to estimate $\text{ var }({{\hat{\varvec{\theta }}}})$ is

1. Fit the model to the sample and calculate ${{\hat{\varvec{\theta }}}}$.
2. Generate bootstrap samples $\{y_{d}^{(b)}: d=1,\ldots ,D\}$, $b=1,\ldots ,B$, from the fitted model.
3. Calculate $${\hat{\varvec{M}}}^{(b)}$$, $$b=1,\ldots ,B$$, and
  $$\begin{aligned} {\overline{\varvec{M}}}=\frac{1}{B}\sum _{b=1}^B{\hat{\varvec{M}}}^{(b)},\quad {\widehat{var}}_B({{\hat{\varvec{M}}}})=\frac{1}{B}\sum _{b=1}^B({\hat{\varvec{M}}}^{(b)}-{\overline{\varvec{M}}})({\hat{\varvec{M}}}^{(b)}-{\overline{\varvec{M}}})^\prime . \end{aligned}$$
4. Output: $$\widehat{\text{ var }}_A({{\hat{\varvec{\theta }}}})=\varvec{H}^{-1}({{\hat{\varvec{\theta }}}})\widehat{\text{ var }}_B({{\hat{\varvec{M}}}})\varvec{H}^{-1}({{\hat{\varvec{\theta }}}})$$.

Alternatively, steps 3 and 4 can be replaced by

3. Fit the model to the bootstrap samples and calculate ${\hat{\varvec{\theta }}}^{(b)}$, $b=1,\ldots ,B$, ${\overline{\varvec{\theta }}}=\frac{1}{B}\sum _{b=1}^B{\hat{\varvec{\theta }}}^{(b)}$.
4. Output: $$\widehat{\text{ var }}_B({{\hat{\varvec{\theta }}}})=\frac{1}{B}\sum _{b=1}^B ({\hat{\varvec{\theta }}}^{(b)}-{\overline{\varvec{\theta }}})({\hat{\varvec{\theta }}}^{(b)}-{\overline{\varvec{\theta }}})^\prime $$.

From this procedure, for a given significance level $\alpha \in (0, 1)$ and the corresponding *t*-value, a model parameter estimate ${\hat{\theta }}_k$ is considered to be significantly different from zero if

$$\begin{aligned} {\hat{\theta }}_k \notin \left( {\hat{\theta }}_k - t_{D-p, 1-\frac{\alpha }{2}} \sqrt{\widehat{\text{ var }}({\hat{\theta }}_k)},\,\, {\hat{\theta }}_k + t_{D-p, 1-\frac{\alpha }{2}} \sqrt{\widehat{\text{ var }}({\hat{\theta }}_k)}\right) . \end{aligned}$$

## Simulation Experiments

This section presents three simulation experiments that are implemented in order to demonstrate the effectiveness of the methodology. We investigate the performance of (i) model parameter estimation, (ii) mean parameter prediction, and (iii) MSE estimation. Note that further simulation results are located in Appendix C of the supplemental material.

### Set Up

A Monte Carlo simulation with $I=500$, $i=1, \ldots , I$, iterations is conducted. We generate a population of *D* domains, where *D* varies over scenarios. For $d=1, \ldots , D$, we define

$$\begin{aligned} \mu _d= \nu _d p_d, \quad y_d \sim \text {Poiss}(\mu _d), \quad p_d = \exp \{\beta _0+\varvec{x}_{1,d} \varvec{\beta }_1 + \varvec{x}_{2,d} \varvec{\beta }_2 + \varvec{u}_{1,d}' \varvec{\beta }_1 + \varvec{u}_{2,d}' \varvec{\beta }_2 + \phi v_d\}, \end{aligned}$$

where $\nu _d = 300$, $\beta _0 = -4$ $\varvec{\beta }_1 = (0.5, 0.5)'$, $\varvec{\beta }_2 = - \varvec{\beta }_1$, and $\phi = 0.3$, as well as $v_d \sim N(0,1)$. Accordingly, we have an intercept and four covariates that are measured with error. Note that the random effects are generated in every iteration individually. The unbiased covariate predictors are drawn from uniform distributions according to $x_{dj} \sim U(1.0,1.4)$, $j=1, \ldots , 4$, and held fixed over all Monte Carlo iterations. The covariate measurement errors $\varvec{u}_d = (\varvec{u}_{1,d}', \varvec{u}_{2,d}')'$ are drawn in each iteration individually according to

$$\begin{aligned} \varvec{u}_d \sim N_{4}({\varvec{0}}, \varvec{\Sigma }_d), \quad \varvec{\Sigma }_d = \begin{pmatrix} \sigma _{1,d}^2 &{} \quad \sigma _{12,d} &{} \quad \sigma _{13,d} &{} \quad \sigma _{14,d}\\ \sigma _{21,d} &{} \quad \sigma _{2,d}^2 &{} \quad \sigma _{23,d} &{} \quad \sigma _{24,d}\\ \sigma _{31,d} &{} \quad \sigma _{32,d} &{} \quad \sigma _{3,d}^2 &{} \quad \sigma _{34,d}\\ \sigma _{41,d} &{} \quad \sigma _{42,d} &{} \quad \sigma _{34,d} &{} \quad \sigma _{4,d}^2\\ \end{pmatrix} , \quad d=1, \ldots , D, \end{aligned}$$

where $\sigma _{j,d}^2 \sim U(0.05, 0.15)$, $\sigma _{jk,d} = \rho _{jk} \sigma _{j,d}^2 \sigma _{k,d}^2$, $\rho _{jk} =0.5$ for $j=1$ and $k=2,3,4$, as well as $\rho _{jk}=-0.3$ for $j=2,3$ and $k=3,4$, $j \ne k$. Overall, we consider four simulation scenarios arising from the four different values for *D*. The scenarios are defined as follows.

**Table 1:** Overview of simulation scenarios

Scenario 1	Scenario 2	Scenario 3	Scenario 4
$D=50$	$D=75$	$D=100$	$D=125$

The simulation is conducted with the statistic software package R. The objective is to estimate the mean parameter $\mu _d$, $d=1, \ldots , D$. We compare the following fitting methods:

- **PM**: maximum likelihood Laplace method for the Poisson mixed model, with the R function glmer.
- **MEPM**: methods of moments for the measurement error Poisson mixed model, with own programming.

### Results of Model Parameter Estimation

This section studies the behavior of the MM algorithm in Sect. 4.1. We consider MSE and bias as performance measures. For parameters $\theta \in \{\beta _0, \varvec{\beta }_1, \varvec{\beta }_2, \phi \}$, they are given by

$$\begin{aligned} MSE(\theta ) = \frac{1}{I} \sum _{i=1}^I ({\hat{\theta }}^{(i)} - \theta )^2, \quad BIAS(\theta ) = \frac{1}{I} \sum _{i=1}^I ({\hat{\theta }}^{(i)} - \theta ). \end{aligned}$$

Note that since $\varvec{\beta }_1 = (\beta _1, \beta _2)'$ with $\beta _1 = \beta _2$, and $\varvec{\beta }_2 = (\beta _3, \beta _4)'$ with $\beta _3=\beta _4$, we average the performance measures for each of for the sake of a compact presentation. Table 2 presents the MSE results, and Table 3 presents the bias results. We start with the regression parameters $\varvec{\beta }= (\beta _0, \varvec{\beta }_1', \varvec{\beta }_2')'$. It can be seen that the MEPM model outperforms the PM model in every scenario and for all elements of $\varvec{\beta }$. The MSE of the estimates obtained from the MM approach are always smaller than those obtained from the maximum likelihood Laplace method under the PM model. The additional information obtained from the anticipation of the covariate measurement errors allows for efficiency gains in these settings. The effect is particular evident when looking at the intercept $\beta _0$. Here, the standard PM model cannot clearly identify the contribution of $\beta _0$ to the functional description of the target variable, which causes excessive variation in the respective estimates. The MEPM model, on the other hand, shows good performance on that regard. For the bias of the regression parameter estimates, we have mixed results. This is due to the fact that measurement error distributions are symmetric around zero. There is no clear tendency which of the two approaches obtains better results. However, when looking at the overall efficiency (in terms of the MSE), the MEPM model is better.

**Table 2:** Mean squared error of model parameter estimation

Scenario	Method	$MSE(\beta _0)$	$MSE(\varvec{\beta }_1)$	$MSE(\varvec{\beta }_2)$	$MSE(\phi )$
1	PM	34.5890	0.4450	0.4625	0.0148
1	MEPM	1.3244	0.3223	0.3202	0.0276
2	PM	33.7888	0.3095	0.2914	0.0069
2	MEPM	1.2405	0.2270	0.2410	0.0173
3	PM	34.4403	0.2551	0.2311	0.0048
3	MEPM	1.3900	0.2506	0.2292	0.0051
4	PM	32.9951	0.1715	0.1805	0.0034
4	MEPM	0.9233	0.1531	0.1623	0.0101

**Table 3:** Bias of model parameter estimation

Scenario	Method	$BIAS(\beta _0)$	$BIAS(\varvec{\beta }_1)$	$BIAS(\varvec{\beta }_2)$	$BIAS(\phi )$
1	PM	5.7220	0.0116	$-$0.0199	$-$0.0511
1	MEPM	0.0710	$-$0.0271	$-$0.0039	$-$0.1055
2	PM	5.6690	$-$0.0044	0.0202	$-$0.0191
2	MEPM	0.0587	$-$0.0420	0.0197	$-$0.0737
3	PM	5.7485	$-$0.0152	$-$0.0033	$-$0.0123
3	MEPM	0.0389	$-$0.0164	$-$0.0037	$-$0.0147
4	PM	5.6555	0.0122	0.0081	$-$0.0066
4	MEPM	$-$0.0416	0.0004	0.0182	$-$0.0513

We now consider the standard deviation parameter estimates. Here, the PM model obtains more efficient results. By looking at the bias, we see that the MEPM approach underestimates the true random effect standard deviation. This is likely due to the additional anticipation of the covariate measurement errors. The method seems to attribute some of the random effect variance to the uncertainty stemming from the auxiliary data, which causes the underestimation tendency and the overall MSE inefficiency. However, we will see in the next section that the predictions obtained under the MEPM model are superior to those under the PM model regardless of this effect.

### Results of Mean Parameter Prediction

This section studies the behavior of the EBP under the area-level MEPM model presented in Sect. 3.2 against the EBP derived by Boubeta et al. (2016) under the area-level Poisson mixed model. We consider relative mean squared error (RMSE), relative root mean squared error (RRMSE), absolute bias (ABIAS), as well as relative absolute bias (RABIAS) as performance measures. They are given as follows:

$$\begin{aligned}&RMSE_{d} = \left( \frac{1}{I} \sum _{i=1}^I (\mu _d^{(i)} - {\hat{\mu }}_d^{(i)})^2 \right) ^{1/2}, \quad RRMSE_d = \frac{RMSE_d}{{\bar{\mu }}_d}, \quad {\bar{\mu }}_d = \frac{1}{I} \sum _{i=1}^I \mu _d^{(i)},\\&ABIAS_d = \frac{1}{I} \sum _{i=1}^I |\mu _d^{(i)} - {\hat{\mu }}_d^{(i)}|, \quad RABIAS_d = \frac{ABIAS_d}{{\bar{\mu }}_d}. \end{aligned}$$

We further define the proportion of efficient predictions (PoEP)

$$\begin{aligned} PoEP = \frac{1}{D} \sum _{d=1}^D \mathbbm {1}_{(RMSE_d < RMSE_d^*)} \cdot 100\%, \end{aligned}$$

as well as the subsequent aggregated measures

$$\begin{aligned}&RMSE = \frac{1}{D} \sum _{d=1}^D RMSE_d, \quad ABIAS = \frac{1}{D} \sum _{d=1}^D ABIAS_d,\\&RRMSE = \frac{1}{D} \sum _{d=1}^R RRMSE_d \cdot 100, \quad RABIAS = \frac{1}{D} \sum _{d=1}^D RABIAS_d \cdot 100\%, \end{aligned}$$

to allow for a more compact presentation. Here, the term $\mathbbm {1}_{(RMSE_d < RMSE_d^*)}$ yields the value 1 when the respective method has achieved a smaller RMSE than the alternative, and 0 else. Table 4 presents the results of mean parameter prediction.

**Table 4:** Performance of mean parameter prediction

Scenario	Method	*RMSE*	*RRMSE*	*ABIAS*	*RABIAS*	*PoEP*
1	PM Model	1.6575	28.6726	1.2896	22.5440	0.00
1	MEPM Model	1.5813	27.3569	1.2323	21.5249	100.00
2	PM Model	1.6146	27.6157	1.2553	21.4650	0.00
2	MEPM Model	1.5684	26.8232	1.2281	21.0016	100.00
3	PM Model	1.5806	27.0935	1.2251	20.9966	4.00
3	MEPM Model	1.5501	26.5669	1.2076	20.6966	96.00
4	PM Model	1.5474	26.7783	1.1975	20.7312	8.00
4	MEPM Model	1.5214	26.3288	1.1879	20.5686	92.00

**Fig. 1:**
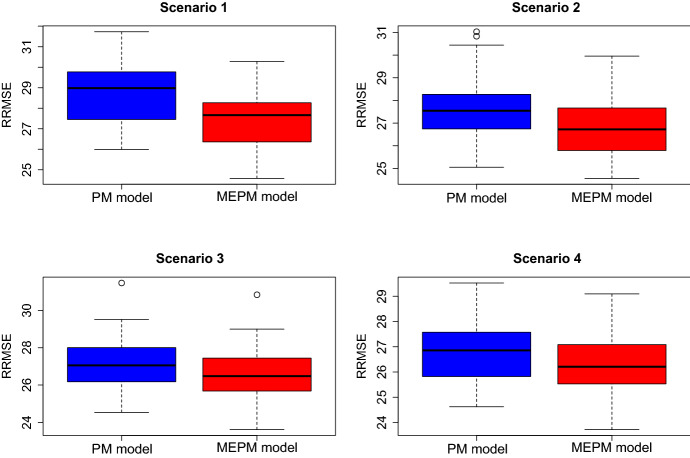
RRMSE of mean parameter prediction

We see that the EBP under the MEPM model outperforms the predictor under the PM model in all scenarios and for all considered performance measures. Further, by looking at the measure *PoEP*, we see that the MEPM predictions are more efficient than the PM predictions in $100\%$ of the domains for Scenario 1 and 2, and in over $90\%$ for Scenario 3 and 4. The anticipation of the covariate measurement errors leads to a significant improvement of the domain count predictions, which is in line with the theoretical developments of Sect. 3. This is further visualized in Fig. 1. It contains boxplots of the measure $RRMSE_d$ over all domains per scenario. The results of the PM model are displayed in blue, while the results of the MEPM model are depicted in red. It becomes evident that both the boxes and the median of the $RRMSE_d$-values are visibly lower under the MEPM model than under the PM model. The strongest efficiency gains are obtained under the scenario with a smaller number of domains. This is important with respect to the empirical application in Sect. 6, where the number of domains is small as well.

### Results of Mean Squared Error Estimation

This section studies the performance of the parametric bootstrap algorithm for MSE estimation in Sect. 3.3. For this, we use the Monte Carlo MSE obtained measured in the simulation experiment as approximation to the real MSE. That is to say, we define

$$\begin{aligned} MSE_{d} = \frac{1}{I} \sum _{i=1}^I (\mu _d^{(i)} - {\hat{\mu }}_d^{(i)})^2, \quad mse_d^* = \frac{1}{I} \sum _{i=1}^{I} mse_d^{*(i)}, \end{aligned}$$

where $mse_d^{*(i)}$ is an MSE estimate obtained for the *d*-th domain in the *i*-th Monte Carlo iteration. Further, we consider absolute bias and relative absolute bias as performance measures, which are given by

$$\begin{aligned} ABIAS_d = \frac{1}{I} \sum _{i=1}^{I} |mse_d^{*(i)} - MSE_d|, \quad RABIAS_d = \frac{ABIAS_d}{MSE_d}\cdot 100\%. \end{aligned}$$

**Fig. 2:**
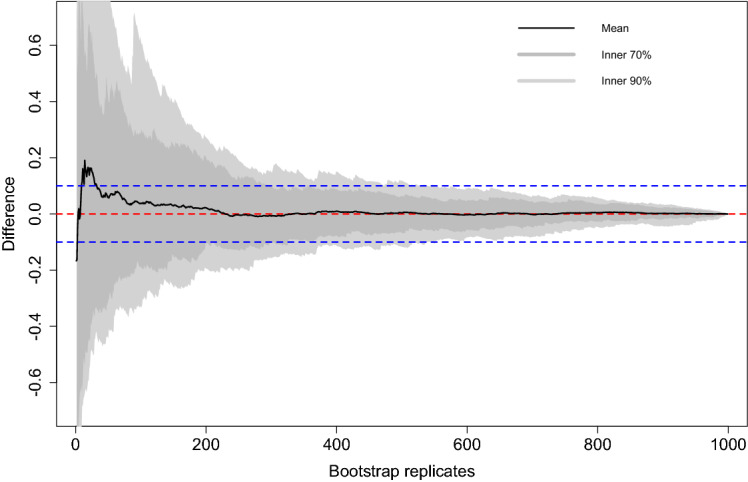
Convergence behavior of MSE estimators per domain

As in the previous two simulation experiments, we use aggregated performance measures

$$\begin{aligned} mse^* = \frac{1}{D} \sum _{d=1}^D mse_d^*, \quad ABIAS = \frac{1}{D} \sum _{d=1}^D ABIAS_d, \quad RABIAS = \frac{1}{D} \sum _{d=1}^D RABIAS_d \end{aligned}$$

for a compact presentation. We choose $B=300$ for the number of bootstrap replicates used for MSE estimation. Table 5 presents the corresponding simulation results and further visualized in Fig. 2. It can be seen that the parametric bootstrap yields plausible results. There is a slight tendency for underestimation for scenarios where the number of domains is small. However, this tendency decreases when increasing the number of domains, although at a rather slow rate. This is because increasing *D* also increases the number of unknown MSE values with to be estimated under new domain-specific measurement error distributions. Furthermore, recall the comment on the MSE estimator’s bias at the end of Sect. 3.3. Depending on the application, researcher may consider using double bootstrap approaches in order to improve MSE estimates. However, with a relative absolute bias of roughly $10\%$, the MSE estimates are relatively accurate given the fact that a GLMM with measurement errors is used for prediction.

**Table 5:** Simulation results for MSE estimation

Scenario	*MSE*	$mse^*$	*ABIAS*	*RABIAS*
1	2.4674	2.2443	0.2643	10.2388
2	2.4741	2.3078	0.2726	10.6629
3	2.4156	2.3800	0.2575	10.4314
4	2.3876	2.2987	0.2312	9.6469

Figure 2 studies the convergence behavior of the parametric bootstrap for MSE estimation. It shows the distribution of the difference $mse_d^*(B) - mse_d^*(1000)$, $d=1, \ldots ,D$, when the parametric bootstrap estimator is calculated based on $B \in \{1, \ldots , 1000\}$ replicates.

The light gray band displays the inner $90\%$ of differences over all domains, while the gray band marks the inner $70\%$. The mean is plotted by the black line. The blue dashed lines mark $10\%$ differences, and the red dashed line displays $0\%$ difference. We see that the mean stabilizes at about $B=250$ iterations. Accordingly, with the $B=300$ we employed in the simulation, the results are generally stable and allow for an evaluation of the overall MSE level in practice. However, in case regional analysis shall be conducted where MSE estimates are compared between domains, a higher number of iterations is required. This is due to the additional uncertainty resulting from the covariate measurement errors. The inner $70\%$ of MSE estimates are within the $10\%$ difference range for $B=400$ , and the inner $90\%$ only for $B=600$ or higher. That is to say, if regional analysis between domains in terms of MSE estimates shall be conducted, we recommend at least $B=600$.

## Regional Prevalence Estimation of MDD

This section presents the application of the MEPM model in Sect. 3 to the data and inferential problem described in Sect. 2. Let us first recall that Sect. 3.1 distinguishes between covariates that are measured without error ${\tilde{\varvec{x}}}_{0,d}$, and covariates that are measured with error ${\tilde{\varvec{x}}}_{1,d}$. In our application, the variables $\{\textit{Domain size}, \textit{Sex} \}$ are measured without error. The first is retrieved from administrative records, and the second is a dummy variable. Their values can be included directly in the MEPM model. For the remaining covariates $\{\textit{Depr. treatment}, \textit{SES score} \}$, we use the GEDA data to obtain unbiased predictors $\varvec{x}_{1,d}$ of the true covariate values that are statistically related to the sample counts $y_d$. For this, we produce Horvitz–Thompson-type direct estimates for ${\tilde{\varvec{x}}}_{1,d}$ using the sample observations and survey weights of GEDA. Against the background of Sect. 3, we note that the Horvitz–Thompson estimator is unbiased and asymptotically normal distributed (Hájek, 1960; Berger, 1998; Chen and Rao, 2007). Accordingly, it satisfies the distribution assumptions with regards to the errors stated in Eq. (1). It further allows us to calculate the domain error covariance matrices $\varvec{\Sigma }_d$, $d=1, \ldots ,D$, which are required in order to account for the measurement errors according to the developments in Sect. 3. For a given covariate $x_{dk}$ in domain $U_d$ that is measured with error, the error variance $\text{ var }(u_{dk})$, $k=1, \ldots , p_1$, is equivalent to the variance of the respective Horvitz–Thompson estimator $\text{ var }(x_{dk})$. The error covariances are obtained from (Wood, 2008)

$$\begin{aligned} \text{ cov }(u_{dj}, u_{dk}) = \text{ cov }(x_{dj}, x_{dk}) = \frac{1}{2}\left( \text{ var }(x_{dj}) + \text{ var }(x_{dk}) - \text{ var }(x_{dj}-x_{dk})\right) , \end{aligned}$$

for $d=1, \ldots , D$ and $j,k=1, \ldots , p_1$ as well as $j \ne k$. Accordingly, for the final area-level MEPM model it holds that $y_d |_{v_d} \sim \text {Poiss}(\mu _d)$, where

$$\begin{aligned} \log \mu _d = \log \nu _d + {\tilde{\varvec{x}}}_{0,d} \varvec{\beta }_0 + \varvec{x}_{1,d} \varvec{\beta }_1 + \varvec{u}_d' \varvec{\beta }_1 + \phi v_d, \quad d=1, \ldots , D. \end{aligned}$$

We perform variance estimation and significance testing for the model parameters as described in Sect. 4.2. In addition to that, we estimate the MSE of the resulting EBPs according to the parametric bootstrap algorithm presented in Sect. 3.3. For both operations, we choose $B=1000$ for the number of bootstrap replicates.

Similarly to Sect. 5, we distinguish between the results of (i) model parameter estimation, (ii) domain prevalence estimation, and (iii) uncertainty estimation. We start with model parameter estimation. The results obtained from fitting the MEPM model as described in the last subsection are summarized in Table 6. It displays the estimated values, the standard deviations, the *p* values with respect to the hypothesis tests $H_0: \theta _k = 0$, $k=1, \ldots , p+1$, as well as 90$\%$ confidence intervals. Note that the last parameter *RE Std.-Dev.* refers to the random effect standard deviation $\phi$. We see that estimated values of the regression parameters are plausible. The covariate *Depr. treatment* is positively linked to the target variable, as could be expected from its definition. The covariates *Sex - male* and *SES score* are negatively associated with the sample counts. This is in line with the literature concerning depressive disorders, as discussed in Sect. 2. The only noticeable variable is *Domain size*, whose coefficient is not significantly different from zero. However, we included it as fixed effect not for explanatory power, but for stability as substitute for an intercept. Thus, we did not expect the covariate to contribute relevantly to the variation of the counts.

**Table 6:** Results of model parameter estimation

Parameter	Estimate	Std.-Dev.	*p* value	$90\%$ CI
*Domain size*	$-$0.00061	0.00134	0.64804	($-$0.00281, 0.00159)
*Sex - male*	$-$0.46819	0.18321	0.01060	($-$0.76957, $-$0.16681)
*Depr. treatment*	1.68745	0.90707	0.06284	(0.19532, 3.17958)
*SES score*	$-$0.25319	0.04167	0.00000	($-$0.32173, $-$0.18464)
*RE Std.-Dev.*	0.76266	0.09162	–	(0.61195, 0.91337)

For the remaining model parameters, it becomes evident that they are significantly different from zero on at least a $90\%$ confidence level. We note that testing significance in the presence of measurement errors is particularly hard. On the one hand, the errors may draw the regression parameter estimates toward zero. On the other hand, anticipating covariate uncertainty tends to increase the variance of regression parameter estimation. Furthermore, the random effects are likely to be masked by the additional variation stemming from measurement errors, as both model components assume normal distributions with expectation zero. Nevertheless, we find all model parameters (expect for the sample size) to be significant in the presented application. Not only the regression parameters, but also that the random effect standard deviation is significantly different from zero. This implies that the random effects are clearly statistically visible in the distribution of the target variable after accounting for the measurement errors.

We continue with domain prevalence estimation. The MDD sample prevalence observed in the SOEP for 2011 is $6.0\%$, where the sex-specific prevalences are $4.2\%$ (male) and $7.8\%$ (female). The MDD prevalence for the German population as estimated by the MEPM model is $6\%$ with a $90\%$ confidence interval of ($5.7\%$, $6.3\%$). This is in line with the results of reference studies, for instance by Busch et al. (2013). They used the German health interview and examination survey for adults from 2008 to 2011 to estimate the 12-month prevalence of diagnosed MDD. The authors reported a total prevalence of $6\%$ for adult Germans. In general, a methodologically sound comparison of the model predictions to external sources is difficult. Many empirical studies do not report MDD prevalence in particular, but depression in a broader sense that also includes lighter forms, such as depressive mood or states of low spirit. On that note, the public health reporting system of Germany stated the combined 12-month prevalence of depression and depressive moods for 2012 at $8\%$ (Gesundheitsberichtserstattung des Bundes 2020). However, with regards to Busch et al. (2013), they explicitly distinguished between diagnosed MDD and lighter forms. Accordingly, the overall level of our prevalence estimates is plausible.

The male prevalence estimated by the MEPM model is $4.2\%$ with a $90\%$ confidence interval of ($3.6\%$, $4.8\%$). Likewise, the female prevalence is estimated at $7.7\%$ with an interval of ($7.1\%$, $8.3\%$). The prevalence for each sex as well as the individual age groups is displayed in Table 7. It contains the respective estimate of the MEPM model parameter (Estimate), a corresponding $90\%$ confidence interval (MEPM 90-CI), the prevalence observed in the sample (Sample), as well as a sample-based $90\%$ confidence interval (Sample 90-CI). We see that the highest MDD prevalence is found in middle-aged females, that is, from 45 to 64 years old. We further see that in the groups of elderly, the MDD frequency overall decreases. These tendencies has also been found by Busch et al. (2013) as well as Robert Koch Institute (2013). Hence, we can conclude that the predicted evolution over the age groups is reasonable. Furthermore, we see that the confidence intervals obtained by MEPM are much smaller than their sample-based counterparts.

**Table 7:** Estimated sex- and age-specific MDD prevalence

Age group	Sex	Estimate	MEPM 90-CI	Sample	Sample 90-CI
18 - 44	Combined	$5.0\%$	($4.5\%$, $5.5\%$)	$4.9\%$	($3.4\%$, $6.5\%$)
18 - 44	Male	$3.4\%$	($2.8\%$, $4.1\%$)	$3.4\%$	($2.0\%$, $4.7\%$)
18 - 44	Female	$6.4\%$	($5.5\%$, $7.2\%$)	$6.3\%$	($4.6\%$, $8.0\%$)
45 - 64	Combined	$8.2\%$	($7.8\%$, $8.6\%$)	$8.4\%$	($6.6\%$, $10.3\%$)
45 - 64	Male	$5.8\%$	($5.3\%$, $6.3\%$)	$6.1\%$	($4.4\%$, $7.7\%$)
45 - 64	Female	$10.3\%$	($9.7\%$, $11.0\%$)	$10.5\%$	($8.5\%$, $12.6\%$)
65+	Combined	$4.2\%$	($3.6\%$, $4.7\%$)	$4.0\%$	($2.4\%$, $5.6\%$)
65+	Male	$2.7\%$	($2.1\%$, $3.4\%$)	$2.5\%$	($1.2\%$, $3.8\%$)
65+	Female	$5.5\%$	($4.6\%$, $6.3\%$)	$5.3\%$	($3.5\%$, $7.2\%$)

**Fig. 3:**
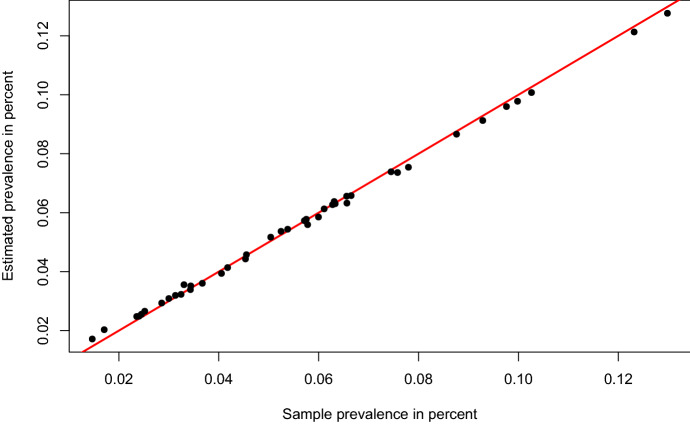
Estimated domain prevalence versus sample domain prevalence

The observed sample prevalence and the estimated prevalence over all domains are compared in Fig. 3. We see that the sample proportions vary randomly around the predicted proportions of the model. This is in line with the distribution assumptions of the MEPM model. It indicates that there is no systematic error in the prediction behavior of the proposed method. We further see that there are no larger deviations, which implies that the MM algorithm has achieved a good fitting performance based on the covariates.

**Fig. 4:**
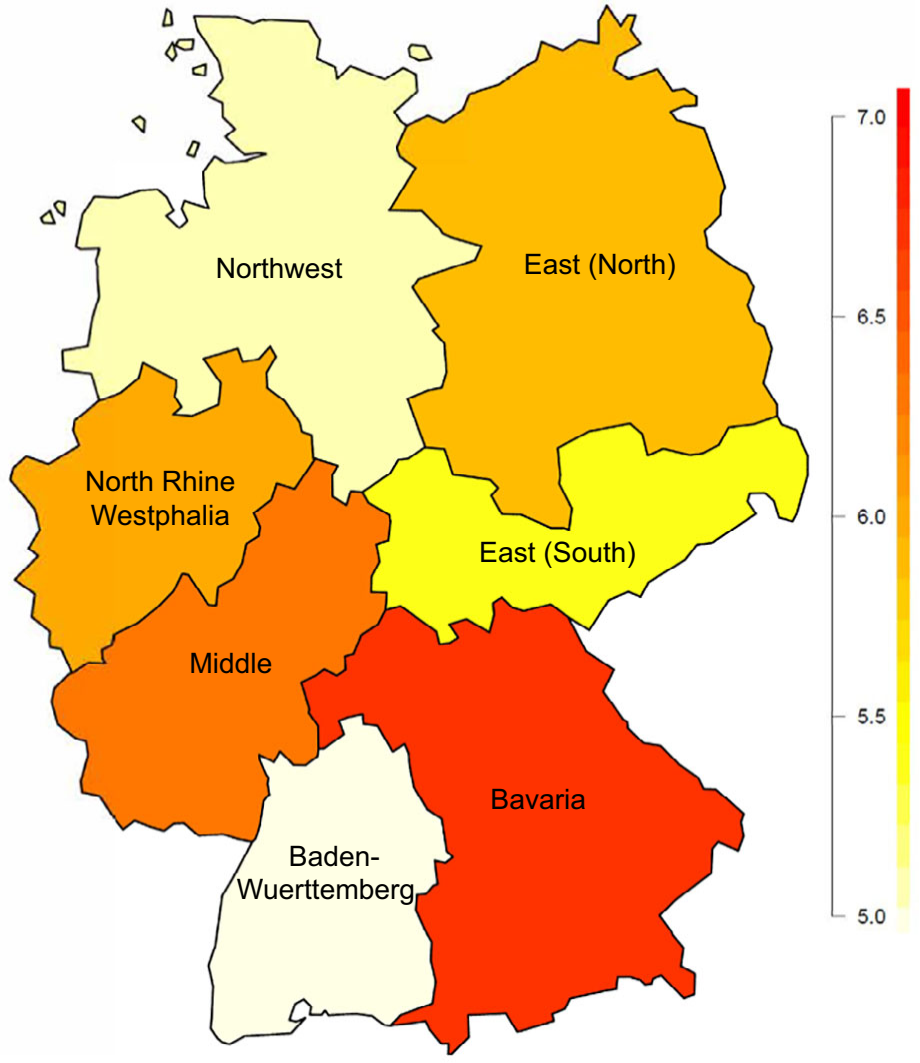
Estimated regional prevalence

Let us now investigate the spatial distribution of MDD in Germany. The corresponding estimates are presented in Fig. 4. It displays a heat map of the MDD prevalence over the seven Nielsen regions of Germany. Males and females are jointly considered in this graph. We see that the highest prevalence can be found in Bavaria. This is in line with the results of Melchior et al. (2014), who found that the highest depression prevalence in Germany for 2014 is located in Bavaria. However, please note that their findings are based on insurance claims of the statutory health insurance company BKK. Therefore, these results are not directly comparable to our survey-based estimates due to several selectivity issues. See Burgard et al. (2019) for further insights on this problem. With respect to the remaining regions, our results display low prevalences in Baden-Württemberg and in Northwest.

**Fig. 5:**
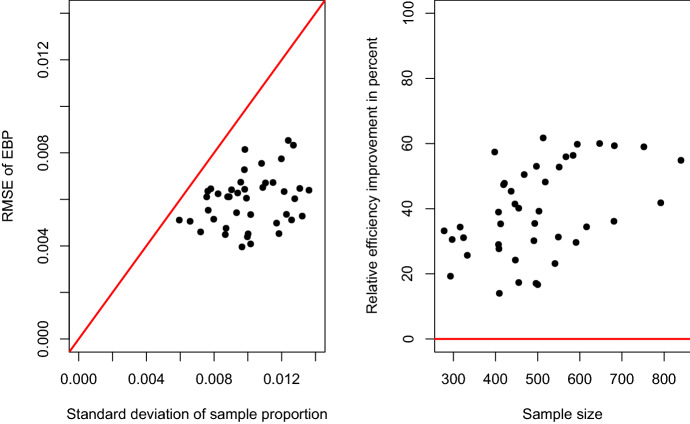
Results of uncertainty estimation

We finally address uncertainty estimation. For this, we compare the results of the parametric bootstrap estimator for MSE estimation in Sect. 3.3 with the uncertainty of the sample prevalence. The results are summarized in Fig. 5. On the left-hand side, the estimated RMSEs of the EBP is plotted against the standard deviations of the sample proportions. The red line indicates the equality of both uncertainty measures. We see that the dots are all located under the line. This implies that the standard deviations of the sample proportions are always larger than the RMSEs of the EBP. On the right-hand side, the relative efficiency improvement

$$\begin{aligned} RE_d=100\,\frac{SD({\tilde{p}}_d) - rmse^*({\hat{p}}_d)}{ SD({\tilde{p}}_d)}, \quad d=1, \ldots , 42, \end{aligned}$$

of the MEPM EBP over the simple sample proportion estimate is depicted. Here, $rmse^*({\hat{p}}_d)$ denotes the estimated RMSE based on the parametric bootstrap sample in domain *d*, and $SD({\tilde{p}}_d)$ is the standard deviation of the sample proportion. We see that the efficiency gains range between $14\%$ and $61\%$. This is also in line with the results presented in Table 7, where the sample-based confidence intervals where visibly larger than those obtained with the MEPM approach. This underlines the efficiency advantage of the method over a standard sample-based estimation.

## Conclusion

This paper proposed a novel model-based approach to estimate MDD prevalence on regional levels using uncertain data. The newly introduced method is an area-level Poisson mixed model that accounts for measurement errors in auxiliary information. With this feature, it is the first GLMM in SAE that allows for erroneous covariate records. The EBP under the model was derived and a parametric bootstrap algorithm for MSE estimation was presented. We further stated a MM fitting algorithm for model parameter estimation. The effectiveness of the methodology was established in several simulation experiments.

The approach was applied to obtained regional MDD prevalence estimation in Germany. The application presents a prevalence map over the seven Nielsen regions of Germany, where men and women are considered together. The map shows the highest prevalence in Bavaria and Midde, which is in line with previous findings in the scientific literature. With respect to the rest of the regions, Baden-Württemberg and the North-West have a low prevalence and the East (South), East (North), and North Rhine-Westphalia are in an intermediate position. This type of disaggregated information allows a better understanding of the spatial distribution of health problems.

The proposed methodology enjoys attractive features from both a theoretical and practical perspective. We start with the theoretical aspects. The derived EBP is an empirical version of the BP, which marks the domain proportion predictor with minimum MSE, in the class of unbiased predictors, under the statistical setting our model considers. Furthermore, the presented MM approach is consistent in model parameter estimation, which is indeed a desirable property for any estimator. With regards to practice, the MEPM model is specified on aggregated data, which is much more likely to be available due to less restrictive privacy regulations. This holds in particular for data on sensitive topics, such as MDD. Further, the approach shows robustness in settings that are quite common in empirical research. We rarely have a census or register data on mental disorders. Accordingly, the vast majority of area-level records in this research field are in fact survey-based estimates and, thus, subject to errors.

A drawback of the method is that it can only be fitted with exclusively area-level data if the respective variances and covariances of the auxiliary variable predictors are known. Otherwise, these terms have to be estimated from the respective survey data as well. This would then require the use of unit-level (person-level) data in addition to the area-level records. However, even if this is necessary, the MEPM model is still more practical than any other model-based unit-level approach. In order to quantify the EBP of a domain quantity under a GLMM, it is typically required to have covariate observations for every person within the target population (Hobza et al. 2018). That is to say, we would need to have unit-level data not only from the sampled individuals, but also from the non-sampled individuals. Such information is rarely available in practice, which makes the MEPM model an attractive alternative for these applications.

In addition to these aspects, the general concept of accounting for measurement errors as presented has a much broader range of applications. Beside the prediction of domain parameters, the methodology may also be used to analyze the outcomes of clinical studies or field experiments. Here, researchers might be interested in the structural relation between several variables, but need to account for measurement interference due to external circumstances. In that case, a corresponding measurement error approach can be used to fit parametric regression models that quantify these relations under the inherent uncertainty.

A related but different problem from error covariates is the incorrect model specification. This is a relevant topic for model-based SAE. Jiang et al. (2011) introduced the OBPs under Fay-Herriot models and under nested error regression models. Chen et al. (2015) extended the OBP approach to area-level Poisson mixed models. These authors show that OBPs are robust with respect to model misspecifications. The extension of the OBP approach to the measurement error Fay-Herriot model of Ybarra and Lohr (2008), or to the measurement error GLMMs, is thus desirable for applications to real data. However, it is not a straightforward task and it will deserve future research.

## Supplementary Information

Below is the link to the electronic supplementary material.

Supplementary material 1 (pdf 320 KB)

## Declarations

### Data availability

The census data on domain sizes used in this study are freely available at the website of the German Federal Statistical Office. The target variable sample data from the survey SOEP are available on demand at Deutsches Institut für Wirtschaftsforschung (German Institute for Economic Research). The auxiliary variable sample data from the survey GEDA are available on demand at the Robert-Koch Institute, Germany. The sample data are protected under national privacy regulations. The authors of this article have no legal permission to share the data with any third party.
