# Supplementary material for: On the Use of Aggregate Survey Data for Estimating Regional Major Depressive Disorder Prevalence
Source: Psychometrika. 2021 Sep 6;87(1):344–68. doi: 10.1007/s11336-021-09808-8 (PMC9021105; doi:10.1007/s11336-021-09808-8)
Supplement: Supplementary file 1 — Supplementary material 1 (pdf 320 KB) [file 11336_2021_9808_MOESM1_ESM.pdf]

On the use of aggregate survey data for estimating regional major depressive disorder prevalence
Supplemental material

Domingo Morales[1], Joscha Krause[2], and Jan Pablo Burgard[2]

[1] *Operations Research Center, University Miguel Hernández de Elche, Spain*
[2] *Department of Economic and Social Statistics, Trier University, Germany*

August 14, 2021

A Newton-Raphson algorithm for the MM method

This section derives the components of the Newton-Raphson algorithm for solving system of nonlinear MM equations. The updating formula is

$$\boldsymbol{\theta}^{(r+1)} = \boldsymbol{\theta}^{(r)} - \boldsymbol{H}^{-1}(\boldsymbol{\theta}^{(r)})\boldsymbol{f}(\boldsymbol{\theta}^{(r)}),$$

where $\theta_1 = \beta_1, \dots, \theta_p = \beta_p$, $\theta_{p+1} = \phi$ and

$$\boldsymbol{\theta} = \underset{1\leq k\leq p+1}{\text{col}}(\theta_k), \quad \boldsymbol{f}(\boldsymbol{\theta}) = \underset{1\leq k\leq p+1}{\text{col}}(f_k(\boldsymbol{\theta})), \quad \boldsymbol{H}(\boldsymbol{\theta}) = \left(\frac{\partial f_a(\boldsymbol{\theta})}{\partial \theta_b}\right)_{a,b=1,\dots,p+1}.$$

Recall that the moment generating function of the univariate Gaussian $y \sim N(\mu, \sigma^2)$ is

$$\psi(t|\mu,\sigma^2) = E\big[\exp\{ty\}\big] = \int_R e^{ty} f(y|\mu,\sigma^2)\, dy = \exp\big\{\mu t + \frac{1}{2}\sigma^2 t^2\big\},$$

and that the moment generating function of the multivariate Gaussian $\boldsymbol{y} \sim N_{p_1}(\boldsymbol{\mu}, \boldsymbol{\Sigma})$ is

$$\Psi(\boldsymbol{t}|\boldsymbol{\mu},\boldsymbol{\Sigma}) = E\big[\exp\{\boldsymbol{t}'\boldsymbol{y}\}\big] = \int_{R^{p_1}} \exp\big\{\boldsymbol{t}'\boldsymbol{y}\big\} f(\boldsymbol{y}|\boldsymbol{\mu},\boldsymbol{\Sigma})\, d\boldsymbol{y} = \exp\big\{\boldsymbol{t}'\boldsymbol{\mu} + \frac{1}{2}\boldsymbol{t}'\boldsymbol{\Sigma}\boldsymbol{t}\big\}.$$

We need to calculate the expectations appearing in $\boldsymbol{f}(\boldsymbol{\theta})$ as well as its partial derivatives. We start with the first p MM equations that correspond to the regression parameters. The expectation (first moment) of y_d is calculated according to

$$\begin{aligned}
E_\theta[y_d] &= E_{u,v}\big[E_\theta[y_d|\boldsymbol{u},\boldsymbol{v}]\big] = E_{u,v}[\nu_d p_d] = E_{u,v}\Big[\nu_d \exp\Big\{\boldsymbol{x}_d\boldsymbol{\beta} + \boldsymbol{\beta}'\boldsymbol{u}_d + \phi v_d\Big\}\Big] \\
&= \int_{R^{p_1+1}} \nu_d \exp\Big\{\boldsymbol{x}_d\boldsymbol{\beta} + \boldsymbol{\beta}'\boldsymbol{u}_d + \phi v_d\Big\} f(v_d) f(\boldsymbol{u}_d)\, dv_d\, \boldsymbol{u}_d \\
&= \nu_d \exp\{\boldsymbol{x}_d\boldsymbol{\beta}\}\Big(\int_R \exp\{\phi v_d\} f(v_d)\, dv_d\Big)\Big(\int_{R^{p_1}} \exp\{\boldsymbol{\beta}'\boldsymbol{u}_d\} f(\boldsymbol{u}_d)\, \boldsymbol{u}_d\Big) \\
&= \nu_d \exp\{\boldsymbol{x}_d\boldsymbol{\beta}\}\psi(\phi|0,1)\Psi(\boldsymbol{\beta}|\boldsymbol{0},\boldsymbol{\Sigma}_d) = \nu_d \exp\{\boldsymbol{x}_d\boldsymbol{\beta}\}\exp\big\{\frac{1}{2}\phi^2\big\}\exp\Big\{\frac{1}{2}\boldsymbol{\beta}_1'\boldsymbol{\Sigma}_d\boldsymbol{\beta}_1\Big\} \\
&= \nu_d \exp\Big\{\boldsymbol{x}_d\boldsymbol{\beta} + \frac{1}{2}\phi^2 + \frac{1}{2}\boldsymbol{\beta}_1'\boldsymbol{\Sigma}_d\boldsymbol{\beta}_1\Big\}.
\end{aligned}$$

Based on these developments, we can conclude that

$$f_k(\boldsymbol{\theta}) = \sum_{d=1}^{D} \nu_d \exp\Big\{\boldsymbol{x}_d\boldsymbol{\beta} + \frac{1}{2}\phi^2 + \frac{1}{2}\boldsymbol{\beta}_1'\boldsymbol{\Sigma}_d\boldsymbol{\beta}_1\Big\} x_{dk} - \sum_{d=1}^{D} y_d x_{dk}, \quad k = 1, \dots p.$$

The derivatives of $E_\theta[y_d]$ are given by

$$\begin{aligned}
\frac{\partial E_\theta[y_d]}{\partial \beta_k} &= \nu_d \exp\Big\{\boldsymbol{x}_d\boldsymbol{\beta} + \frac{1}{2}\Big(\phi^2 + \boldsymbol{\beta}_1'\boldsymbol{\Sigma}_d\boldsymbol{\beta}_1\Big)\Big\}(x_{dk} + \boldsymbol{\delta}_k'\boldsymbol{\Sigma}_d\boldsymbol{\beta}_1), \quad k = 1, \dots, p_1, \\
\frac{\partial E_\theta[y_d]}{\partial \beta_k} &= \nu_d \exp\Big\{\boldsymbol{x}_d\boldsymbol{\beta} + \frac{1}{2}\Big(\phi^2 + \boldsymbol{\beta}_1'\boldsymbol{\Sigma}_d\boldsymbol{\beta}_1\Big)\Big\} x_{dk}, \quad k = p_1 + 1, \dots, p, \\
\frac{\partial E_\theta[y_d]}{\partial \phi} &= \nu_d \exp\Big\{\boldsymbol{x}_d\boldsymbol{\beta} + \frac{1}{2}\Big(\phi^2 + \boldsymbol{\beta}_1'\boldsymbol{\Sigma}_d\boldsymbol{\beta}_1\Big)\Big\}\phi.
\end{aligned}$$

Regarding the second moment, the expectation of y_d^2 is $E_\theta[y_d^2] = E_{u,v}\big[E_\theta[y_d^2|\boldsymbol{u}, \boldsymbol{v}]\big]$, where

$$E_\theta[y_d^2|\boldsymbol{u}, \boldsymbol{v}] = \text{var}_\theta[y_d|\boldsymbol{u}, \boldsymbol{v}] + E_\theta^2[y_d|\boldsymbol{u}, \boldsymbol{v}] = \nu_d p_d + \nu_d^2 p_d^2.$$

Accordingly, it follows that

$$E_\theta[y_d^2] = E_{u,v}\big[E_\theta[y_d^2|\boldsymbol{u}, \boldsymbol{v}]\big] = \int_{R^{p_1+1}} \nu_d p_d f(v_d) f(\boldsymbol{u}_d)\, dv_d\, d\boldsymbol{u}_d + \int_{R^{p_1+1}} \nu_d^2 p_d^2 f(v_d) f(\boldsymbol{u}_d)\, dv_d\, d\boldsymbol{u}_d.$$

We have

$$\begin{aligned}
I_2 &= \int_{R^{p_1+1}} p_d^2 f(v_d) f(\boldsymbol{u}_d)\, dv_d\, d\boldsymbol{u}_d = \int_{R^{p_1+1}} \exp\Big\{2\boldsymbol{x}_d\boldsymbol{\beta} + 2\boldsymbol{\beta}'\boldsymbol{u}_d + 2\phi v_d\Big\} f(v_d) f(\boldsymbol{u}_d)\, dv_d\, d\boldsymbol{u}_d \\
&= \exp\big\{2\boldsymbol{x}_d\boldsymbol{\beta}\big\}\psi(2\phi|0,1)\Psi(2\boldsymbol{\beta}|\boldsymbol{0}, \boldsymbol{\Sigma}_d) = \exp\big\{2\boldsymbol{x}_d\boldsymbol{\beta}\big\}\exp\big\{2\phi^2\big\}\exp\Big\{2\boldsymbol{\beta}_1'\boldsymbol{\Sigma}_d\boldsymbol{\beta}_1\Big\} \\
&= \exp\Big\{2\boldsymbol{x}_d\boldsymbol{\beta} + 2\phi^2 + 2\boldsymbol{\beta}_1'\boldsymbol{\Sigma}_d\boldsymbol{\beta}_1\Big\}.
\end{aligned}$$

and

$$E_\theta[y_d^2] = \nu_d \exp\big\{\boldsymbol{x}_d\boldsymbol{\beta} + \frac{1}{2}\boldsymbol{\beta}_1'\boldsymbol{\Sigma}_d\boldsymbol{\beta}_1 + \frac{1}{2}\phi^2\big\} + \nu_d^2 \exp\big\{2\boldsymbol{x}_d\boldsymbol{\beta} + 2\boldsymbol{\beta}_1'\boldsymbol{\Sigma}_d\boldsymbol{\beta}_1 + 2\phi^2\big\}.$$

We can conclude that

$$f_{p+1}(\boldsymbol{\theta}) = \sum_{d=1}^{D}\Big\{\nu_d \exp\Big\{\boldsymbol{x}_d\boldsymbol{\beta} + \frac{1}{2}\phi^2 + \frac{1}{2}\boldsymbol{\beta}_1'\boldsymbol{\Sigma}_d\boldsymbol{\beta}_1\Big\} + \nu_d^2 \exp\Big\{2\boldsymbol{x}_d\boldsymbol{\beta} + 2\phi^2 + 2\boldsymbol{\beta}_1'\boldsymbol{\Sigma}_d\boldsymbol{\beta}_1\Big\}\Big\} - \sum_{d=1}^{D} y_d^2.$$

The derivatives of $E_\theta[y_d^2]$ are

$$\begin{aligned}
\frac{\partial E_\theta[y_d^2]}{\partial \beta_k} &= \nu_d \exp\Big\{\boldsymbol{x}_d\boldsymbol{\beta} + \frac{1}{2}\phi^2 + \frac{1}{2}\boldsymbol{\beta}_1'\boldsymbol{\Sigma}_d\boldsymbol{\beta}_1\Big\}(x_{dk} + \boldsymbol{\delta}_k'\boldsymbol{\Sigma}_d\boldsymbol{\beta}_1) \\
&+ 2\nu_d^2 \exp\Big\{2\boldsymbol{x}_d\boldsymbol{\beta} + 2\phi^2 + 2\boldsymbol{\beta}_1'\boldsymbol{\Sigma}_d\boldsymbol{\beta}_1\Big\}(x_{dk} + \boldsymbol{\delta}_k'\boldsymbol{\Sigma}_d\boldsymbol{\beta}_1), \quad k = 1, \dots, p_1, \\
\frac{\partial E_\theta[y_d^2]}{\partial \beta_k} &= \nu_d \exp\Big\{\boldsymbol{x}_d\boldsymbol{\beta} + \frac{1}{2}\phi^2 + \frac{1}{2}\boldsymbol{\beta}_1'\boldsymbol{\Sigma}_d\boldsymbol{\beta}_1\Big\} x_{dk} \\
&+ 2\nu_d^2 \exp\Big\{2\boldsymbol{x}_d\boldsymbol{\beta} + 2\phi^2 + 2\boldsymbol{\beta}_1'\boldsymbol{\Sigma}_d\boldsymbol{\beta}_1\Big\} x_{dk}, \quad k = p_1 + 1, \dots, p, \\
\frac{\partial E_\theta[y_d^2]}{\partial \phi} &= \nu_d \exp\Big\{\boldsymbol{x}_d\boldsymbol{\beta} + \frac{1}{2}\phi^2 + \frac{1}{2}\boldsymbol{\beta}_1'\boldsymbol{\Sigma}_d\boldsymbol{\beta}_1\Big\}\phi + 4\nu_d^2 \exp\Big\{2\boldsymbol{x}_d\boldsymbol{\beta} + 2\phi^2 + 2\boldsymbol{\beta}_1'\boldsymbol{\Sigma}_d\boldsymbol{\beta}_1\Big\}\phi.
\end{aligned}$$

The elements of the Jacobian matrix are

$$\begin{aligned} H_{rk} &= \frac{\partial f_r(\boldsymbol{\theta})}{\partial \theta_k} = \sum_{d=1}^{D} \frac{\partial E_\theta[y_d]}{\partial \theta_k} x_{dk}, \quad r=1,\ldots,p,\ k=1,\ldots,p+1, \\ H_{p+1k} &= \frac{\partial f_{p+1}(\boldsymbol{\theta})}{\partial \theta_k} = \sum_{d=1}^{D} \frac{\partial E_\theta[y_d^2]}{\partial \theta_k}, \quad k=1,\ldots,p+1. \end{aligned}$$

B Consistency of the MM estimator

This section deals with the consistency of the MM estimator as $D \to \infty$. It presents the adaptations of Lemma 1 and Theorem 1 of Jiang (1998) to the new MEPM model. Jiang (1998) gives a proof for GLMMs where the moments that appear in the MM equations must be calculated by the Monte Carlo method. In the case of the Poisson distribution, these moments can be calculated explicitly and this simplifies the proofs because it is not necessary to impose regularity conditions for the Monte Carlo approach.

Note that for models with intercept, the MM equation 1 is

$$\sum_{d=1}^{D} E_\theta[y_d] = \sum_{d=1} y_d.$$

As

$$\left(\sum_{d=1}^{D} y_d\right)^2 = \sum_{d=1}^{D} y_d^2 + \sum_{d\neq\ell}^{D} y_d y_\ell,$$

we can follow Jiang (1998) and substitute the MM equation $p+1$ by

$$\sum_{d\neq\ell}^{D} E_\theta[y_d y_\ell] = \sum_{d\neq\ell}^{D} y_d y_\ell.$$

Let $X \xrightarrow{L^2} 0$ denote L_2-convergence, i.e. $E[X^2] \longrightarrow 0$. Denote $\eta_{0d} = \eta_d(\boldsymbol{\theta}_0)$, $d=1,\ldots,D$, where $\boldsymbol{\theta}_0$ is the true parameter. Define the subset of R^4

$$Q = \big\{(1,0,0,0),(1,1,0,0),(2,1,0,0),(2,2,0,0),(1,1,1,1)\big\}.$$

We first give a lemma that states the convergence of equations (3) and (4) of the main text to zero. In Lemma 1, condition (1) states the requirements for the expectations of the products of derivatives of $b(\eta) = e^\eta$. The conditions (2) and (3) specify the orders of the normalizing constants a_{Dk} and b_D. The conclusions appear in (4) and (5).

Lemma 1. *Suppose that*

$$K_D = \max_{1\le d,\ell,d',\ell'\le D} \max_{(a,b,c,r)\in Q} E_{\theta_0}\left[e^{a\eta_{0d}} e^{b\eta_{0\ell}} e^{c\eta_{0d'}} e^{r\eta_{0\ell'}}\right] < \infty, \quad \limsup_{D\to\infty} K_D < \infty, \tag{1}$$

and that the sequences $\{a_{Dk}\}$, $k=1,\ldots,p$, and $\{b_D\}$ satisfy

$$\varepsilon_{D,k} = \max_{1\le d\le D} \left\{ \frac{1}{a_{Dk}^2} \max\left\{ \sum_{d=1}^{D} x_{dk}^2, \sum_{d=1}^{D}\sum_{\ell=1}^{D} |x_{dk} x_{d\ell}| \right\} \right\} \underset{D\to\infty}{\longrightarrow} 0, \quad k=1,\ldots,p. \tag{2}$$

and

$$\varepsilon_{D,p+1} = \max_{1\le d\le D} \frac{D^4}{b_D^2} \underset{D\to\infty}{\longrightarrow} 0. \tag{3}$$

Then

$$\frac{1}{a_{Dk}} \sum_{d=1}^{D} x_{dk}\big(y_d - E_{\theta_0}[y_d]\big) \underset{D\to\infty}{\overset{L^2}{\longrightarrow}} 0, \quad k = 1, \dots p, \tag{4}$$

and

$$\frac{1}{b_D} \sum_{d\neq \ell}^{D} \big(y_d y_\ell - E_{\theta_0}[y_d y_\ell]\big) \underset{D\to\infty}{\overset{L^2}{\longrightarrow}} 0. \tag{5}$$

Proof. We use the notation $E_\theta[\cdot]$ and $E[\cdot]$ for expectations with respect to distributions depending and not depending on $\boldsymbol{\theta}$, respectively. More concretely, we use $E_\theta[\cdot]$ for the distributions of y_d and $y_d|(v_d, \boldsymbol{u}_d)$, and $E[\cdot]$ for the distribution of $(v_d, \boldsymbol{u}_d')$.

For the equation k, $k = 1, \dots, p$, it holds that

$$E_k = E_{\theta_0}\bigg[\bigg(\sum_{d=1}^{D} x_{dk}\big(y_d - E_{\theta_0}[y_d]\big)\bigg)^2\bigg] = E_{\theta_0}\bigg[\bigg(\sum_{d=1}^{D} x_{dk}\big(y_d - \dot{b}(\eta_{0d})\big) + \sum_{d=1}^{D} x_{dk}\big(\dot{b}(\eta_{0d}) - E_{\theta_0}[y_d]\big)\bigg)^2\bigg].$$

We recall that

$$E_{\theta_0}[y_d] = E\big[E_{\theta_0}[y_d|v_d, \boldsymbol{u}_d]\big] = E\big[e^{\eta_{0d}}\big] = E\big[\dot{b}(\eta_{0d})\big]$$

and that

$$(a+b)^2 = a^2 + b^2 + 2ab = 2a^2 + 2b^2 - (a^2 + b^2 - 2ab) = 2a^2 + 2b^2 - (a-b)^2 \le 2a^2 + 2b^2.$$

Take

$$a = \sum_{d=1}^{D} x_{dk}\big(y_d - \dot{b}(\eta_{0d})\big), \quad b = \sum_{d=1}^{D} x_{dk}\big(\dot{b}(\eta_{0d}) - E_{\theta_0}[y_d]\big).$$

Then, we have

$$E_k \le 2\bigg\{E_{\theta_0}\bigg[\bigg(\sum_{d=1}^{D} x_{dk}\big(y_d - \dot{b}(\eta_{0d})\big)\bigg)^2\bigg] + E\bigg[\bigg(\sum_{d=1}^{D} x_{dk}\big(\dot{b}(\eta_{0d}) - E_{\theta_0}[y_d]\big)\bigg)^2\bigg] = 2(S_1 + S_2).$$

The first summand is

$$S_1 = \sum_{d=1}^{D} x_{dk}^2 E_{\theta_0}\Big[\big(y_d - \dot{b}(\eta_{0d})\big)^2\Big] + \sum_{d\neq\ell}^{D} x_{dk} x_{d\ell} E_{\theta_0}\Big[\big(y_d - \dot{b}(\eta_{0d})\big)\big(y_\ell - \dot{b}(\eta_{0\ell})\big)\Big].$$

The first expectations are

$$E_{\theta_0}\Big[\big(y_d - \dot{b}(\eta_{0d})\big)^2\Big] = E\Big[E_{\theta_0}\Big[\big(y_d - E_{\theta_0}[y_d|v_d, \boldsymbol{u}_d]\big)^2 \big| v_d, \boldsymbol{u}_d\Big]\Big] = E\big[\ddot{b}(\eta_{0d})\big].$$

Because of the independence, conditioned to $\boldsymbol{v}$ and $\boldsymbol{u}$, the second expectations are

$$E_{\theta_0}\Big[\big(y_d - \dot{b}(\eta_{0d})\big)\big(y_\ell - \dot{b}(\eta_{0\ell})\big)\Big] = E\Big[E_{\theta_0}\Big[\big(y_d - E_{\theta_0}[y_d|v_d, \boldsymbol{u}_d]\big)\big(y_\ell - E_{\theta_0}[y_\ell|v_\ell, \boldsymbol{u}_\ell]\big) \big| v, \boldsymbol{u}\Big]\Big]$$

$$= E\Big[E_{\theta_0}\Big[y_d - E_{\theta_0}[y_d|v_d, \boldsymbol{u}_d] \big| v_d, \boldsymbol{u}_d\Big] E_{\theta_0}\Big[y_\ell - E_{\theta_0}[y_\ell|v_\ell, \boldsymbol{u}_\ell] \big| v_\ell, \boldsymbol{u}_\ell\Big]\Big] = 0.$$

Therefore, the first summand takes the form

$$S_1 = \sum_{d=1}^{D} x_{dk}^2 E\big[\ddot{b}(\eta_{0d})\big].$$

The second summand is

$$S_2 = \sum_{d=1}^{D} x_{dk}^2 E\Big[\big(\dot{b}(\eta_{0d}) - E\big[\dot{b}(\eta_{0d})\big]\big)^2\Big] + \sum_{d\neq\ell}^{D} x_{dk}x_{d\ell}E_{\theta_0}\Big[\big(\dot{b}(\eta_{0d}) - E\big[\dot{b}(\eta_{0d})\big]\big)\big(\dot{b}(\eta_{0\ell}) - E\big[\dot{b}(\eta_{0\ell})\big]\big)\Big].$$

The expectations are

$$\begin{aligned} E\Big[\big(\dot{b}(\eta_{0d}) - E\big[\dot{b}(\eta_{0d})\big]\big)^2\Big] &= \operatorname{var}\big(\dot{b}(\eta_{0d})\big), \\ E\Big[\big(\dot{b}(\eta_{0d}) - E\big[\dot{b}(\eta_{0d})\big]\big)\big(\dot{b}(\eta_{0\ell}) - E\big[\dot{b}(\eta_{0\ell})\big]\big)\Big] &= \operatorname{cov}\big(\dot{b}(\eta_{0d}), \dot{b}(\eta_{0\ell})\big). \end{aligned}$$

Therefore, the second summand takes the form

$$S_2 = \sum_{d=1}^{D}\sum_{\ell=1}^{D} x_{dk}x_{d\ell}\operatorname{cov}\big(\dot{b}(\eta_{0d}), \dot{b}(\eta_{0\ell})\big).$$

Going back to E_k, we have

$$\begin{aligned} E_k &\leq 2\Big\{\sum_{d=1}^{D} x_{dk}^2 E\big[\ddot{b}(\eta_{0d})\big] + \sum_{d=1}^{D}\sum_{\ell=1}^{D} x_{dk}x_{d\ell}\operatorname{cov}\big(\dot{b}(\eta_{0d}), \dot{b}(\eta_{0\ell})\big)\Big\} \\ &\leq 2K_D\Big\{\sum_{d=1}^{D} x_{dk}^2 + \sum_{d=1}^{D}\sum_{\ell=1}^{D} |x_{dk}x_{d\ell}|\Big\}. \end{aligned}$$

Thus (4) follows by (1) and (2).

Concerning the equation $p+1$, it holds that

$$\begin{aligned} E_{p+1} &= \sum_{d\neq\ell}^{D}\big(y_d y_\ell - E_{\theta_0}[y_d y_\ell]\big) = \sum_{d\neq\ell}^{D}(y_d - \dot{b}(\eta_{0d}))(y_\ell - \dot{b}(\eta_{0\ell})) + \sum_{d\neq\ell}^{D}\dot{b}(\eta_{0d})(y_\ell - \dot{b}(\eta_{0\ell})) \\ &+ \sum_{d\neq\ell}^{D}(y_d - \dot{b}(\eta_{0d}))\dot{b}(\eta_{0\ell}) + \sum_{d\neq\ell}^{D}\big\{\dot{b}(\eta_{0d})\dot{b}(\eta_{0\ell}) - E_{\theta_0}[y_d y_\ell]\big\} = I_1 + I_2 + I_3 + I_4. \end{aligned}$$

In what follow, we apply the following property. If $X_1, \ldots, X_n$ are independent with $E[X_i] = 0$ and $E[X_i^4] < \infty$, and if $\boldsymbol{A} = \big(a_{ij}\big)_{1\leq i,j\leq n}$ is a symmetric matrix, then

$$\begin{aligned} E\Big[\Big(\sum_{i=1}^{n}\sum_{j=1}^{n} a_{ij}X_iX_j - \sum_{i=1}^{n} a_{ii}E[X_i^2]\Big)^2 &= \sum_{i=1}^{n} a_{ii}^2\operatorname{var}(X_i^2) + 2\sum_{i\neq j}^{n} a_{ij}^2 E[X_i^2]E[X_j^2] \\ &\leq 2\sum_{i\neq j}^{n} a_{ij}^2 E[X_i^2]E[X_j^2]. \end{aligned}$$

Using this fact and recalling that

$$E_{\theta_0}[y_d|v_d,\boldsymbol{u}_d]=\dot b(\eta_{0d})=e^{\eta_{0d}},\quad \operatorname{var}_{\theta_0}[y_d|v_d,\boldsymbol{u}_d]=\ddot b(\eta_{0d})=e^{\eta_{0d}},$$

leads to

$$\begin{aligned}
E_{\theta_0}[I_1^2] &= E\Big[E_{\theta_0}\Big[\Big(\sum_{d=1}^D\sum_{\ell=1}^D \delta_{d\ell}(y_d-\dot b(\eta_{0d}))(y_\ell-\dot b(\eta_{0\ell}))\Big)^2\Big|\boldsymbol{v},\boldsymbol{u}\Big]\Big] \\
&\le 2E\Big[\sum_{d\neq\ell}^D E_{\theta_0}\Big[(y_d-\dot b(\eta_{0d}))^2\big|v_d,\boldsymbol{u}_d\Big]E_{\theta_0}\Big[(y_\ell-\dot b(\eta_{0\ell}))^2\big|v_d,\boldsymbol{u}_d\Big]\Big] \\
&= 2\sum_{d\neq\ell}^D E\Big[\ddot b(\eta_{0d})\ddot b(\eta_{0\ell})\Big]=2\sum_{d\neq\ell}^D E\Big[e^{\eta_{0d}}e^{\eta_{0\ell}}\Big]\le 2D(D-1)K_D\le 2D^2K_D,
\end{aligned}$$

where $\delta_{d\ell}$ is the Kronecker's delta.

$$\begin{aligned}
E_{\theta_0}[I_2^2] &= E\Big[E_{\theta_0}\Big[\Big(\sum_{d=1}^D\sum_{\ell=1}^D \delta_{d\ell}\dot b(\eta_{0d})(y_\ell-\dot b(\eta_{0\ell}))\Big)^2\Big|\boldsymbol{v},\boldsymbol{u}\Big]\Big] \\
&\le 2E\Big[\sum_{d\neq\ell}^D \dot b^2(\eta_{0d})E_{\theta_0}\Big[(y_\ell-\dot b(\eta_{0\ell}))^2\big|v_\ell,\boldsymbol{u}_\ell\Big]\Big] \\
&= 2\sum_{d\neq\ell}^D E\Big[\dot b^2(\eta_{0d})\ddot b(\eta_{0\ell})\Big]=2\sum_{d\neq\ell}^D E\Big[e^{2\eta_{0d}}e^{\eta_{0\ell}}\Big]\le 2D(D-1)K_D\le 2D^2K_D.
\end{aligned}$$

Similarly $E_{\theta_0}[I_3^2]\le 2D^2K_D$.

$$\begin{aligned}
E_{\theta_0}[I_4^2] &= E\Big[\Big(\sum_{d\neq\ell}^D\big\{\dot b(\eta_{0d})\dot b(\eta_{0\ell})-E_{\theta_0}[y_dy_\ell]\big\}\Big)^2\Big] \\
&= \sum_{d\neq\ell}^D\sum_{d'\neq\ell'}^D E\Big[\Big\{\dot b(\eta_{0d})\dot b(\eta_{0\ell})-E\big[\dot b(\eta_{0d})\dot b(\eta_{0\ell})\big]\Big\}\Big\{\dot b(\eta_{0d'})\dot b(\eta_{0\ell'})-E\big[\dot b(\eta_{0d'})\dot b(\eta_{0\ell'})\big]\Big\}\Big] \\
&= \sum_{d\neq\ell}^D\sum_{d'\neq\ell'}^D \operatorname{cov}\Big(\dot b(\eta_{0d})\dot b(\eta_{0\ell}),\,\dot b(\eta_{0d'})\dot b(\eta_{0\ell'})\Big)\le\sum_{d\neq\ell}^D\sum_{d'\neq\ell'}^D E\Big[\dot b(\eta_{0d})\dot b(\eta_{0\ell})\dot b(\eta_{0d'})\dot b(\eta_{0\ell'})\Big] \\
&= \sum_{d\neq\ell}^D\sum_{d'\neq\ell'}^D E\Big[e^{\eta_{0d}}e^{\eta_{0\ell}}e^{\eta_{0d'}}e^{\eta_{0\ell'}}\Big]\le D^4K_D.
\end{aligned}$$

Thus (5) follows by (1) and (3). □

Let us define

$$M_{D,k}(\boldsymbol{\theta})=\frac{1}{a_{Dk}}\sum_{d=1}^D x_{dk}E_\theta[y_d],\quad M_{D,p+1}(\boldsymbol{\theta})=\frac{1}{b_D}\sum_{d=1}^D E_\theta[y_d^2],$$

$$\hat{M}_{D,k} = \frac{1}{a_{Dk}} \sum_{d=1}^{D} x_{dk} y_d, \quad \hat{M}_{D,p+1} = \frac{1}{b_D} \sum_{d=1}^{D} y_d^2,$$

$$\tilde{M}_{D,k}(\boldsymbol{\theta}) = \frac{1}{a_{Dk}} \sum_{d=1}^{D} x_{dk} \dot{b}(\eta_d), \quad \tilde{M}_{D,p+1}(\boldsymbol{\theta}) = \frac{1}{b_D} \sum_{d \neq \ell}^{D} \dot{b}(\eta_d) \dot{b}(\eta_\ell),$$

and

$$\boldsymbol{M}_D(\boldsymbol{\theta}) = \big(M_{D,k}(\boldsymbol{\theta})\big)_{1 \le k \le p+1}, \ \hat{\boldsymbol{M}}_D = \big(\hat{M}_{D,k}\big)_{1 \le k \le p+1}, \ \tilde{\boldsymbol{M}}_D(\boldsymbol{\theta}) = \big(\tilde{M}_{D,k}(\boldsymbol{\theta})\big)_{1 \le k \le p+1}.$$

Lemma 1 gives sufficient conditions for

$$\|\hat{\boldsymbol{M}}_D - \boldsymbol{M}_D(\boldsymbol{\theta}_0)\| \xrightarrow[D \to \infty]{L^2} 0$$

Let $\{A_{Dk}\}$, $1 \le k \le p$, and $\{B_D\}$ any sequences such that $A_{Dk} \to \infty$ and $B_D \to \infty$ as $D \to \infty$. Let $\hat{\boldsymbol{\theta}} = (\hat{\boldsymbol{\beta}}', \hat{\phi})$ be any $\boldsymbol{\theta} = (\boldsymbol{\beta}', \phi) \in \Theta_D = \big\{\boldsymbol{\theta} : |\beta_k| \le A_{Dk}, 1 \le k \le p; \, 0 < \phi \le B_D\big\}$ satisfying the inequality

$$\big\|\tilde{\boldsymbol{M}}_D(\boldsymbol{\theta}) - \hat{\boldsymbol{M}}_D\big\| \le \delta_D, \tag{6}$$

where $\delta_D \to \infty$ as $D \to \infty$.

The following theorem states the consistency of the MM estimators of the parameters of the MEPM model. The proof follows from Theorem 1 of Jiang (1998) by doing the particularization to the current MEPM model and by noting that:

(1) Jiang (1998) gives a proof for GLMMs with q random effects. Here we consider the Poisson mixed model with one random effect, but we add p random measurement errors. The random errors have a known multivariate normal distribution and are independent of the random effect.

(2) The moments $M_k(\boldsymbol{\theta})$ are calculated analytically and not approximated by the Monte Carlo method, and

(3) The expectations $E[\cdot]$ are taken with respect to the joint distribution of v_d and $\boldsymbol{u}_d$, $d = 1, \ldots, D$, as it was shown in Lemma 1.

Because of (1)-(3), there are not remarkable difficulties in adapting and particularizing the proof of Jiang. We present the sketch of the proof. For more details, see Jiang (1998).

Theorem 1. *Suppose that the conditions of Lemma 1 are satisfied. Let $\boldsymbol{\theta}_0$ be the true parameter and let $\varepsilon_D = \max_{1 \le k \le p+1}\{\varepsilon_{D,k}\}$.*

(a) *If $\varepsilon_D/\delta_D^2 \to \infty$, then $\hat{\boldsymbol{\theta}}$ exists with probability tending to one as $D \to \infty$.*

(b) *If, furthermore, all the first derivatives of the expectations $E_\theta[y_d] = E[\dot{b}(\eta_d(\boldsymbol{\theta}))]$ and $E_\theta[y_d y_\ell] = E[\dot{b}(\eta_d(\boldsymbol{\theta}))\dot{b}(\eta_\ell(\boldsymbol{\theta}))]$, with respect to the components of $\boldsymbol{\theta}$, can be taken under the expectation sign; the quantities*

$$\sup_{\|\boldsymbol{\theta}\| \le B} E\Big[\big(\dot{b}(\eta_d(\boldsymbol{\theta}))\big)^4\Big], \ E\Big[\sup_{\|\boldsymbol{\theta}\| \le B} \ddot{b}(\eta_d(\boldsymbol{\theta}))\Big], \ E\Big[\sup_{\|\boldsymbol{\theta}\| \le B} \ddot{b}(\eta_d(\boldsymbol{\theta}))\big|\dot{b}(\eta_\ell(\boldsymbol{\theta}))\big|\Big], \ d, \ell = 1, \ldots, D,$$

are finite for any $B > 0$*; and*

$$\liminf_{D\to\infty} \inf_{\|\boldsymbol{\theta}-\boldsymbol{\theta}_0\|>\varepsilon} \||\boldsymbol{M}_D(\boldsymbol{\theta}) - \boldsymbol{M}_D(\boldsymbol{\theta}_0)\| > 0$$

for any $\varepsilon > 0$*, then* $\hat{\boldsymbol{\theta}}$ *converges to* $\boldsymbol{\theta}_0$ *with probability tending to one.*

Proof.

(a) By the proof of Lemma 1, we have

$$E_{\theta_0}\big[\|\hat{\boldsymbol{M}}_D - \boldsymbol{M}_D(\boldsymbol{\theta}_0)\|^2\big] = \sum_{k=1}^{p+1} E_k \leq (2p+4)K_D\varepsilon_D \underset{D\to\infty}{\longrightarrow} 0.$$

On the other hand

$$E_{\theta_0}\big[(\tilde{M}_{D,k}(\boldsymbol{\theta}_0) - M_{D,k}(\boldsymbol{\theta}_0))^2\big] = E\Bigg[\bigg(\frac{1}{a_{Dk}}\sum_{d=1}^{D} x_{dk}\big(\dot{b}(\eta_{0d}) - E[\dot{b}(\eta_{0d})]\big)\bigg)^2\Bigg] \leq \frac{K_D}{a_{Dk}^2}\sum_{d\neq\ell}|x_{dk}x_{d\ell}| \leq K_D\varepsilon_D.$$

and similarly

$$E_{\theta_0}\big[(\tilde{M}_{D,p+1}(\boldsymbol{\theta}_0) - M_{D,p+1}(\boldsymbol{\theta}_0))^2\big] \leq \frac{K_D}{b_D^2}D^4 \leq K_D\varepsilon_D.$$

Thus, we have

$$\begin{aligned} P\Big(\|\tilde{\boldsymbol{M}}_D(\boldsymbol{\theta}_0) - \hat{\boldsymbol{M}}_D\| \leq \delta_D\Big) &\leq P\Big(\|\tilde{\boldsymbol{M}}_D(\boldsymbol{\theta}_0) - \boldsymbol{M}_D(\boldsymbol{\theta}_0)\| \leq \frac{\delta_D}{2}\Big) + P\Big(\|\boldsymbol{M}_D(\boldsymbol{\theta}_0) - \hat{\boldsymbol{M}}_D\| \leq \frac{\delta_D}{2}\Big) \\ &\leq [(p+1) + (2p+4)]\frac{K_D\varepsilon_D}{\delta_D} \underset{D\to\infty}{\longrightarrow} 0. \end{aligned}$$

Therefore, (6) holds and $\hat{\boldsymbol{\theta}}$ exists with probability tending to one at $\theta = \theta_0$.

(b) Because of the continuity of $\boldsymbol{M}$ with respect to $\boldsymbol{\theta}$, it enough to prove that for any $\delta > 0$, it holds

$$P\big(\|\boldsymbol{M}_D(\hat{\boldsymbol{\theta}}) - \boldsymbol{M}_D(\boldsymbol{\theta}_0)\| \geq \delta\big) \underset{D\to\infty}{\longrightarrow} 0.$$

We have that

$$\begin{aligned} \|\boldsymbol{M}_D(\hat{\boldsymbol{\theta}}) - \boldsymbol{M}_D(\boldsymbol{\theta}_0)\| &\leq \|\boldsymbol{M}_D(\hat{\boldsymbol{\theta}}) - \tilde{\boldsymbol{M}}_D(\hat{\boldsymbol{\theta}})\| + \|\tilde{\boldsymbol{M}}_D(\hat{\boldsymbol{\theta}}) - \hat{\boldsymbol{M}}_D\| + \|\hat{\boldsymbol{M}}_D - \boldsymbol{M}_D(\boldsymbol{\theta}_0)\| \\ &\leq \sup_{\boldsymbol{\theta}\in\Theta_D} \|\boldsymbol{M}_D(\boldsymbol{\theta}) - \tilde{\boldsymbol{M}}_D(\boldsymbol{\theta})\| + \delta_D + \|\hat{\boldsymbol{M}}_D - \boldsymbol{M}_D(\boldsymbol{\theta}_0)\|. \end{aligned}$$

Because of Lemma 1, the third summand becomes bounded and close to zero as $D \to \infty$. By expanding $\boldsymbol{M}_D(\hat{\boldsymbol{\theta}})$ and $\tilde{\boldsymbol{M}}_D(\hat{\boldsymbol{\theta}})$ in Taylor's series around some $\boldsymbol{\theta}_*$ in a neighborhood of $\boldsymbol{\theta}_0$, we mimic the same steps of the proof of Theorem 1(b) of Jiang (1988) to obtain a bound for the first summand as $D \to \infty$. This fact allows applying the the Chebyshev's inequality for bounded random variables and we get

$$P\big(\|\boldsymbol{M}_D(\hat{\boldsymbol{\theta}}) - \boldsymbol{M}_D(\boldsymbol{\theta}_0)\| \geq \delta\big) \leq \frac{1}{\delta}E\big[\|\boldsymbol{M}_D(\hat{\boldsymbol{\theta}}) - \boldsymbol{M}_D(\boldsymbol{\theta}_0)\|\big] \underset{D\to\infty}{\longrightarrow} 0.$$

This completes the proof. □

C Additional simulation experiments

C.1 Set up

This section presents additional simulation experiments, in which we compare the explicit measurement error modelling implemented by the MEPM model against the simulative approach used by SIMEX. For the latter, we use the R-package *simex* provided by Lederer et al. (2019). Please note that this package does not yet allow to fit generalized linear mixed models with measurement errors. However, it allows for the fitting of generalized linear models with measurement errors (without random effects). Thus, in order to avoid giving SIMEX an unfair disadvantage, we alter the data generating process presented in Section 5 of the main text by setting the random effect standard deviation to zero. Accordingly, we consider the measurement error Poisson (MEP), the standard Poisson (P) model, and the SIMEX approach that uses the standard Poisson as naive model. Since the objective of the paper is the prediction of regional prevalence figures, we focus on mean parameter prediction in the simulation hereafter.

A Monte Carlo simulation with $I = 750$, $i = 1, \ldots, I$, iterations is conducted. We generate a population of D domains, where D varies over scenarios. For $d = 1, ..., D$, we define

$$\mu_d = \nu_d p_d, \quad y_d \sim \text{Poiss}(\mu_d), \quad p_d = \exp\{\beta_0 + \boldsymbol{x}_{1,d}\boldsymbol{\beta}_1 + \boldsymbol{x}_{2,d}\boldsymbol{\beta}_2 + \boldsymbol{u}'_{1,d}\boldsymbol{\beta}_1 + \boldsymbol{u}'_{2,d}\boldsymbol{\beta}_2\},$$

where $\nu_d = 300$, $\beta_0 = -4$ $\boldsymbol{\beta}_1 = (0.5, 0.5)'$, and $\boldsymbol{\beta}_2 = -\boldsymbol{\beta}_1$. Accordingly, we have an intercept and four covariates that are measured with error. Note that the random effects are generated in every iteration individually. The unbiased covariate predictors are drawn from uniform distributions according to $x_{dj} \sim U(1.0, 1.4)$, $j = 1, ..., 4$, and held fixed over all Monte Carlo iterations. The covariate measurement errors $\boldsymbol{u}_d = (\boldsymbol{u}'_{1,d}, \boldsymbol{u}'_{2,d})'$ are drawn in each iteration individually according to

$$\boldsymbol{u}_d \sim N_4(\boldsymbol{0}, \boldsymbol{\Sigma}_d), \quad \boldsymbol{\Sigma}_d = \begin{pmatrix} \sigma^2_{1,d} & \sigma_{12,d} & \sigma_{13,d} & \sigma_{14,d} \\ \sigma_{21,d} & \sigma^2_{2,d} & \sigma_{23,d} & \sigma_{24,d} \\ \sigma_{31,d} & \sigma_{32,d} & \sigma^2_{3,d} & \sigma_{34,d} \\ \sigma_{41,d} & \sigma_{42,d} & \sigma_{34,d} & \sigma^2_{4,d} \end{pmatrix}, \quad d = 1, \ldots, D,$$

where $\sigma^2_{j,d} \sim U(0.05, 0.15)$, $\sigma_{jk,d} = \rho_{jk}\sigma^2_{j,d}\sigma^2_{k,d}$, $\rho_{jk} = 0.5$ for $j = 1$ and $k = 2, 3, 4$, as well as $\rho_{jk} = -0.3$ for $j = 2, 3$ and $k = 3, 4$, $j \neq k$. Just like in Section 5 of the main text, we consider four simulation scenarios arising from the four different values for D. The scenarios are defined as in Table 1 of the main text.

C.2 Results

We consider relative mean squared error (RMSE), relative root mean squared error (RRMSE), absolute bias (ABIAS), as well as relative absolute bias (RABIAS) as performance measures. They are given as follows:

$$RMSE_d = \left(\frac{1}{I}\sum_{i=1}^{I}(\mu_d^{(i)} - \hat{\mu}_d^{(i)})^2\right)^{1/2}, \quad RRMSE_d = \frac{RMSE_d}{\bar{\mu}_d}, \quad \bar{\mu}_d = \frac{1}{I}\sum_{i=1}^{I}\mu_d^{(i)},$$

$$ABIAS_d = \frac{1}{I}\sum_{i=1}^{I}|\mu_d^{(i)} - \hat{\mu}_d^{(i)}|, \quad RABIAS_d = \frac{ABIAS_d}{\bar{\mu}_d}.$$

We further define the subsequent aggregated measures

$$RMSE = \frac{1}{D}\sum_{d=1}^{D} RMSE_d, \quad ABIAS = \frac{1}{D}\sum_{d=1}^{D} ABIAS_d,$$

$$RRMSE = \frac{1}{D}\sum_{d=1}^{R} RRMSE_d \cdot 100, \quad RABIAS = \frac{1}{D}\sum_{d=1}^{D} RABIAS_d \cdot 100\%,$$

to allow for compact presentation. The results are summarized in Table 1 and Figure 1.

Method	Scenario	$RMSE$	$RRMSE$	$ABIAS$	$RABIAS$
P	1	0.7086	19.2049	0.5631	15.4376
SIMEX	1	0.6963	18.8702	0.5548	15.2096
MEP	1	0.5770	15.6044	0.4460	12.2295
P	2	0.6376	17.1027	0.5059	13.5730
SIMEX	2	0.6226	16.7053	0.4958	13.4092
MEP	2	0.5357	14.3590	0.4163	11.1577
P	3	0.5747	15.4733	0.4560	12.2784
SIMEX	3	0.5615	15.1224	0.4471	12.1412
MEP	3	0.5477	14.7210	0.4236	11.3904
P	4	0.5449	14.7829	0.4318	11.7164
SIMEX	4	0.5294	14.3652	0.4212	11.5733
MEP	4	0.4788	12.9803	0.3746	10.1575

Table 1: Results of mean parameter prediction

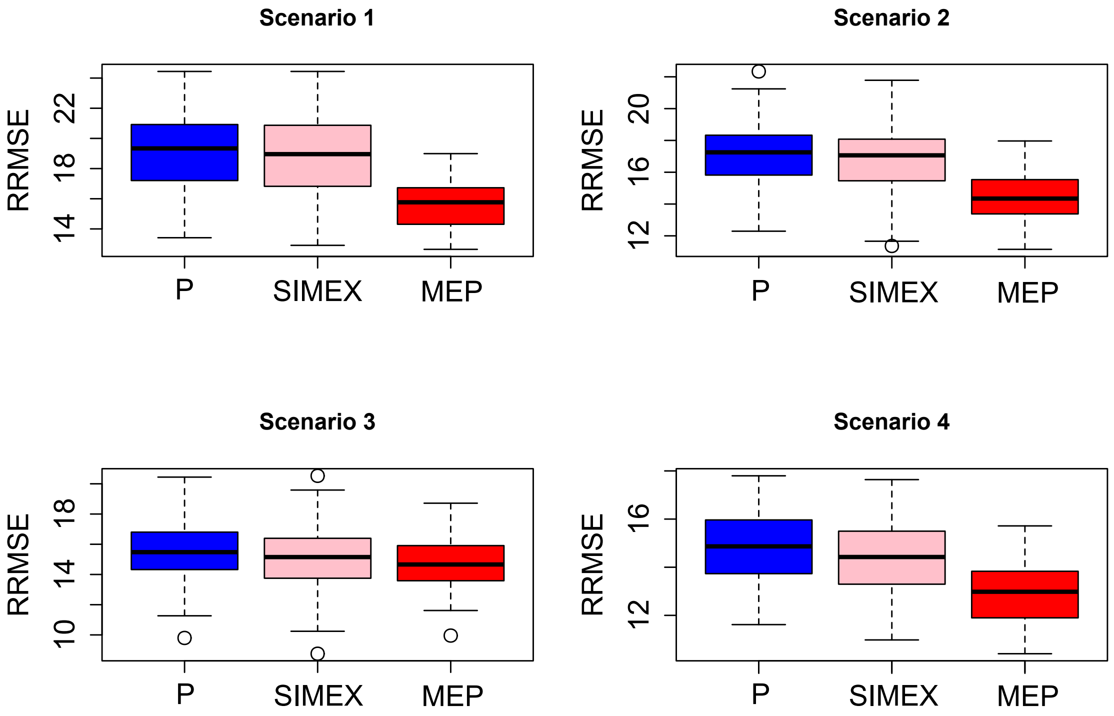

Figure 1: Domain-level mean parameter prediction performance

In Table 1, we see that the SIMEX approach outperforms the standard Poisson model in all scenarios and for all performance measures. This could be expected from theory, since SIMEX has been introduced as an effective and flexible technique to deal with measurement errors. However, the SIMEX approach does not reach the efficiency level of the MEP approach. The latter dominates in all scenarios and all performance measures by a considerable margin. This is likely due to the explicit consideration of the measurement errors in the model equation. All inferential steps are strictly derived under this premise. That is to say, for this particular setting, the proposed MEP approach is the best considered option.

Figure 1 displays the measure $RRMSE_d$ for all considered domains and simulation scenarios. The predictions obtained under the standard Poisson model are marked in blue. The predictions from the SIMEX approach are plotted in light red. And finally, the predictions obtained under the proposed MEP approach are displayed in red. In accordance with Table 1, we see that the MEP approach outperforms the other methods significantly. Its RRMSE on domain-level is always smaller. However, we further see that the range of RRMSE values is also much narrower for the MEP approach. This implies that the method overall obtains more stable results relative to standard Poisson and SIMEX.

References

Jiang, J. (1998). Consistent estimators in generalized linear models. *Journal of the American Statistical Association*, **93**, 720-729.

Lederer, W., Seibold, H., Küchenhoff, H., Lawrence, C., Brondum, R. F. (2019). Package 'simex'. URL: https://cran.r-project.org/web/packages/simex/simex.pdf.
